# Structure of HCMV glycoprotein B in the postfusion conformation bound to a neutralizing human antibody

**DOI:** 10.1038/ncomms9176

**Published:** 2015-09-14

**Authors:** Sumana Chandramouli, Claudio Ciferri, Pavel A. Nikitin, Stefano Caló, Rachel Gerrein, Kara Balabanis, James Monroe, Christy Hebner, Anders E. Lilja, Ethan C. Settembre, Andrea Carfi

**Affiliations:** 1 GSK Vaccines, 45 Sidney Street, Cambridge, Massachusetts 02139, USA; 2 Novartis Institutes for Biomedical Research, Emeryville, California 94608, USA; 3 GSK Vaccines, Via Fiorentina 1, Siena 53100, Italy; 4 Novartis Influenza Vaccines, 45 Sidney Street, Cambridge, Massachusetts 02139, USA

## Abstract

Human cytomegalovirus (HCMV) poses a significant threat to immunocompromised individuals and neonates infected *in utero*. Glycoprotein B (gB), the herpesvirus fusion protein, is a target for neutralizing antibodies and a vaccine candidate due to its indispensable role in infection. Here we show the crystal structure of the HCMV gB ectodomain bound to the Fab fragment of 1G2, a neutralizing human monoclonal antibody isolated from a seropositive subject. The gB/1G2 interaction is dominated by aromatic residues in the 1G2 heavy chain CDR3 protruding into a hydrophobic cleft in the gB antigenic domain 5 (AD-5). Structural analysis and comparison with HSV gB suggest the location of additional neutralizing antibody binding sites on HCMV gB. Finally, immunoprecipitation experiments reveal that 1G2 can bind to HCMV virion gB suggesting that its epitope is exposed and accessible on the virus surface. Our data will support the development of vaccines and therapeutic antibodies against HCMV infection.

## Abstract (web summary)

Cytomegalovirus is a danger to individuals with compromised immune systems and neonates infected *in utero*. Here the authors show the structure of a neutralizing antibody-bound viral fusion protein glycoprotein B, supporting the development of therapeutic antibodies and vaccines.

## Graphical abstract

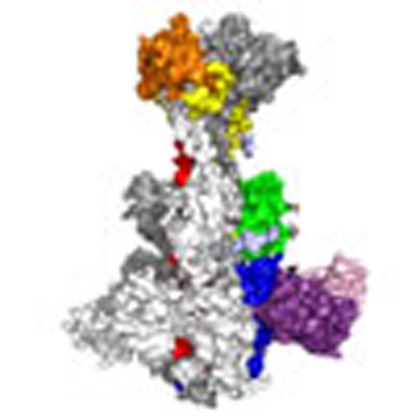

Human cytomegalovirus (HCMV) is a double stranded DNA virus of the β-herpesvirus family. HCMV is the leading cause of congenital and neonatal hearing loss resulting from vertical virus transmission following infection or reactivation of latent virus in pregnant women^1^. In addition, HCMV is one of the most common opportunistic pathogens affecting immunosuppressed transplant patients. Infection of seronegative subjects receiving solid organ transplants from seropositive donors is positively correlated with increased incidence of allograft rejection^2^. Currently, antiviral therapy following transplantation remains the only mode of intervention, often combined with hyperimmune globulin transfusion to help control viremia^3^. Though development of a vaccine against HCMV has been listed as a top priority by the Institute of Medicine^4^,^5^, none has been licensed so far.

The HCMV genome encodes several envelope glycoproteins. Among them, glycoprotein B (gB) and the gH/gL heterodimer are conserved within the herpesvirus families (α, β and γ) forming the core fusion machinery essential for viral entry into cells^6^. HCMV cell entry can be distinguished into two steps, receptor binding and membrane fusion. Receptor-binding function resides within distinct gH/gL-containing complexes: gH/gL/gO for entry into fibroblasts and gH/gL/UL128/UL130/UL131A (Pentamer) for entry into epithelial/endothelial cells^7^,^8^,^9^,^10^. Membrane fusion is mediated by gB, which acts as the fusogen^11^,^12^. How cognate receptor binding of gH/gL complexes triggers the conformational changes in gB required for membrane fusion remains unknown. In the case of herpes simplex virus (HSV), an α-herpesvirus, bimolecular complementation assays have shown evidence for an interaction between gB and gH/gL during cell fusion^13^,^14^. Preliminary co-immunoprecipitation experiments in epithelial cells have also provided some evidence for an interaction between gH/gL and gB in HCMV^15^.

HCMV subunit vaccines incorporating soluble forms of gB have been under development for several years (reviewed in^16^,^17^). Immunization with a gB subunit adjuvanted with MF59 resulted in ∼50% protective efficacy against primary infection in post-partum healthy women^18^,^19^. Similarly, an MF59-adjuvanted gB vaccine led to significant reduction in viremia and shortened antiviral treatment in patients receiving solid organ transplants^20^. Furthermore, in a guinea pig maternal immunization/infection model, a three-dose regimen with a guinea pig CMV gB subunit vaccine adjuvanted with AS01 or AS02 reduced pup mortality by 64–84%, and reduced viremia in infected dams by nearly sixfold^21^. Together these studies confirmed that the gB subunit vaccines were safe and immunogenic, though further improvements in potency and durability of protection were desirable.

Monoclonal antibodies (mAbs) against HCMV have a potential use as an alternative or addition to the current forms of HCMV therapy. Neutralizing antibodies against HCMV gB were shown to inhibit entry of cell-free virus in several cell types (including fibroblasts, endothelial and epithelial cells) and to prevent cell-to-cell spread^22^. Moreover in a recent study, protection from brain pathology in murine cytomegalovirus infected mice was accomplished by passive transfer of a gB-specific monoclonal antibody^23^. A better understanding of the antigenic determinants of gB and biochemical and functional characterization of potent, broadly neutralizing antibodies is invaluable for developing therapeutic antibody candidates and vaccines against HCMV infection.

Crystal structures of HSV and Epstein–Barr Virus (EBV) postfusion gB have shown a close similarity despite the relatively low sequence identity^11^,^24^. These structures revealed that the gB ectodomain has an elongated three-lobed shape and is composed of five structural domains (I–V). In addition, low resolution negative stain electron microscopy (EM) studies of HCMV gB have confirmed a conserved overall architecture for postfusion gB across the three herpesvirus families^25^,^26^,^27^. Finally, structural analysis has revealed a close similarity between herpesvirus gB and the postfusion conformation of the vesicular stomatitis virus G and the baculovirus Gp64 glycoproteins^28^,^29^,^30^ suggesting that recombinant gB spontaneously folds into the postfusion conformation. Despite this progress, the crystal structure of HCMV gB has remained elusive, in part due to the difficulty of obtaining large amounts of homogeneous sample.

EM and mutagenesis studies have located some of the key neutralizing sites in HSV gB^11^,^31^. In HCMV gB, five antigenic domains (AD) have been defined, with neutralizing epitopes mapped to AD-1 (within structural domain IV), AD-2 (within the first 80 N-terminal residues), AD-4 (in structural domain II) and AD-5 (in structural domain I)^32^,^33^. AD-3 resides in the very C terminus of gB beyond the transmembrane region and no neutralizing antibodies targeting this domain have been reported to date. Many human neutralizing antibodies targeting gB have been isolated from IgG-secreting memory B cells from HCMV seropositive individuals^33^,^34^ and shown to neutralize infection of both fibroblasts and epithelial cells in the absence of complement. Several of these antibodies have been found to target AD-4 (SM1–6, SM3-1, SM4–5, SM5-1, SM6-5 and SM11–17) and AD-5 (SM10, 1G2, SM12 and 2C2), and key gB residues critical for binding to mAbs have been identified^35^,^36^. The structure of a complex between the SM5 Fab and an *E.coli*-expressed gB AD-4 domain only (spanning residues 112–132 and 344–438) has also recently been reported^37^.

We have introduced mutations in HCMV gB that allow its secretion as a homogeneous and stable postfusion trimer from mammalian cells. Here we report the crystal structure of this molecule in complex with the 1G2 Fab. The structure provides the first atomic view of HCMV gB and of its interaction with a broadly neutralizing antibody targeting the conserved AD-5 site. These data will guide the design of improved gB-based HCMV vaccines and will support the development of therapeutic antibodies against this medically relevant human pathogen.

## Results

### Generation of a soluble and homogeneous gB ectodomain

**Figure 1:**
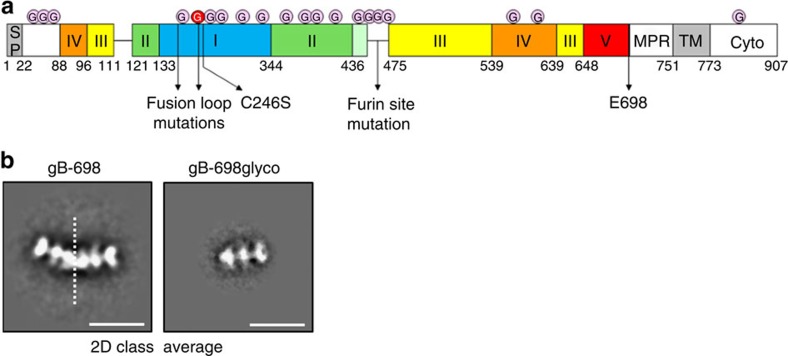
Electron microscopy of gB ectodomain constructs. (**a**) Schematic of full-length gB with domains I–V coloured as in^11^. Mutations introduced in gB ectodomain constructs and glycosylation sites are indicated by arrows and pink circles, respectively. An additional glycosylation site introduced in fusion loop 2 is indicated by a red circle. SP, signal peptide; MPR, membrane proximal region; TM, transmembrane region; Cyto, cytoplasmic domain. E698 indicates the last C-terminal residue in the gB ectodomain constructs. Details of mutations in the fusion loops and furin site are reported in Materials and Methods. (**b**) Two-dimensional class averages of negative stain electron micrographs of gB-698 (left panel) and gB-698glyco (right panel). Images represent projections of molecule side views. Dimer boundary (left panel) is indicated by a dashed line. Scale bar, 200 Å.

The ectodomain of HCMV gB (Merlin strain), residues 1–698, with a 6-His tag at the C terminus was expressed in HEK293S GnTI^−^ cells (Fig. 1a). To increase protein secretion, we mutated three hydrophobic residues in the fusion loops with the corresponding more hydrophilic amino acids from HSV-1 gB (I157H, H158R and W240R). We also mutated the canonical furin cleavage site to decrease protein heterogeneity caused by incomplete processing during expression, as well as the exposed Cys246 (Cys246Ser) to prevent formation of spurious disulfide bonds (Fig. 1a). Despite these changes, size exclusion chromatography (SEC) and negative stain EM (Fig. 1b) revealed that the protein (gB-698) formed dimers of the characteristic three-lobed postfusion trimers^26^,^38^. Analysis of the EM images suggested that dimerization was mediated by the base of the gB trimer, presumably due to intrinsic surface hydrophobicity. Thus, we introduced a glycosylation site in fusion loop-2 (Trp240Asn and Tyr242Thr), predicted to be solvent exposed in the trimer, to interfere with dimerization. EM and SEC confirmed that this construct, gB-698glyco, does not dimerize even at high protein concentration (Fig. 1b).

### EM of HCMV gB bound to 1G2 Fab

**Figure 2:**
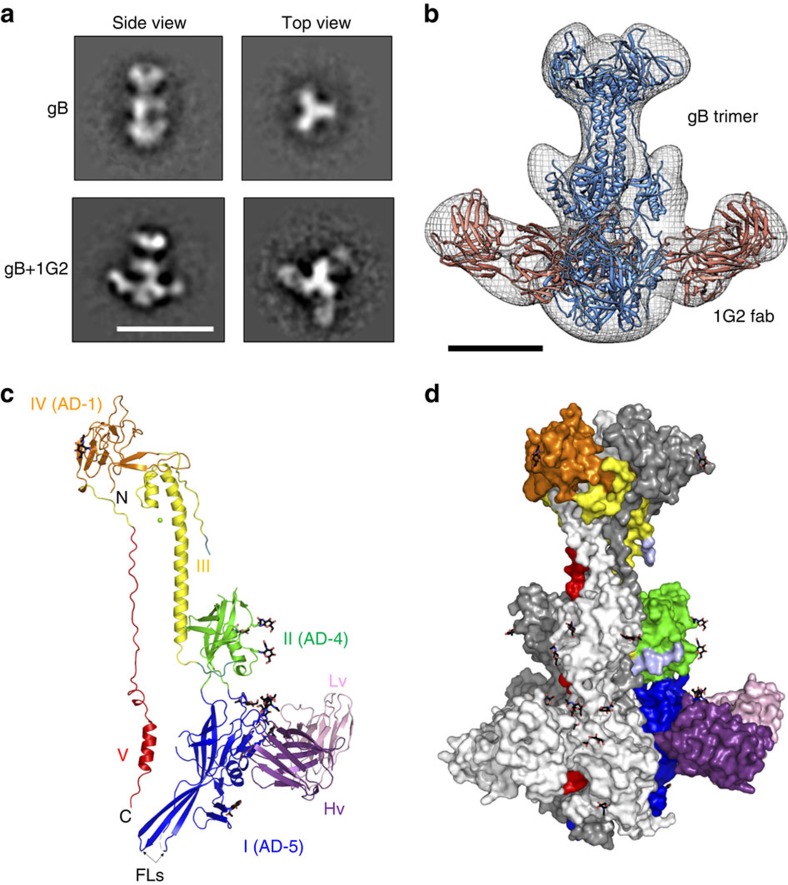
Structure of gB/1G2 complex. (**a**) EM two-dimensional class averages of gB-698glyco (top panels) and gB-698glyco in complex with 1G2 Fab (bottom panels). Scale bar, 200 Å. (**b**) Low resolution three-dimensional reconstruction of gB-698glyco/1G2 Fab complex generated from EM data with homology models of gB and 1G2 fitted in the electron density map. gB trimer is shown in blue and the 1G2 Fab is shown in red. Scale bar, 50 Å. (**c**) Crystal structure of ΔNgB-glyco/1G2 complex (PDB ID: 5C6T). The asymmetric unit contained a single gB monomer bound to 1G2. The domains of gB are coloured as in Fig. 1a. The 1G2 Fab heavy chain is shown in purple and the light chain in light pink. The glycans resolved in the structure are shown in black, and a Mg ion in domain III is shown as a green dot. (**d**) Surface representation of the ΔNgB-glyco/1G2 trimer. One monomer is positioned and coloured as in (**c**) while the other two are represented in white and grey.

EM was used to initially characterize the gB/1G2 Fab complex. Comparison of negative stain two-dimensional class averages of gB-698glyco alone and bound to 1G2 confirmed that all three sites on the gB trimer are occupied by Fab molecules (Fig. 2a and Supplementary Fig. 1A). Moreover, individual particles, extracted from negative-stained micrographs, were used to generate a low resolution three-dimensional reconstruction of the complex at 19 Å, using the crystal structure of HSV gB low pass filtered at 60 Å as initial model. In agreement with previous mutagenesis data^33^,^36^, the EM analysis confirmed that 1G2 binds to structural domain I (residues 134–343; Figs 1a and 2b).

### Structure determination of gB/1G2 Fab complex

Initial attempts at crystallizing gB-698glyco alone or in complex with 1G2 Fab were unsuccessful. We therefore enzymatically deglycosylated the protein with endoglycosidase H and performed *in situ* limited proteolysis with subtilisin E to remove flexible regions that could interfere with crystallization^39^,^40^. This treatment resulted in crystals that diffracted to 4.3 Å resolution. Subsequently, we deleted the first 63 N-terminal residues, shown to be flexible in HSV-1 gB^11^, from gB-698glyco resulting in the ΔNgB-glyco construct (residues 86–698). The deglycosylated ΔNgB-glyco/1G2 Fab complex crystallized readily without need for protease treatment. After screening several crystals, a 3.6-Å resolution dataset was obtained and the structure determined by the molecular replacement method (Table 1). The resulting electron density maps allowed model building for gB and the Fv portion of the Fab (Supplementary Fig. 2). Though crystal packing allows enough space for the constant regions of the Fab heavy and light chains, no electron density was observed for these even at the end of refinement, suggesting that they are flexibly linked to the variable region and adopt different orientations in the crystals.

### HCMV gB structural analysis and comparison with related gBs

The overall domain organization of postfusion HCMV gB is similar to HSV and EBV gBs. Five structural domains are distributed across the length of the molecule (Fig. 2c,d), with structural domains I and II adopting PH-domain-like folds. Domain I also harbours bipartite hydrophobic fusion loops exposed at the base of the molecule. Domain III forms a long central coiled-coil helix leading up to domain IV, the crown at the top of the molecule. Domain V, composed of the very C-terminal residues of the ectodomain, zips down the length of the molecule nestled between the other two protomers of the trimer, terminating close to the fusion loops at the base of the molecule.

**Figure 3:**
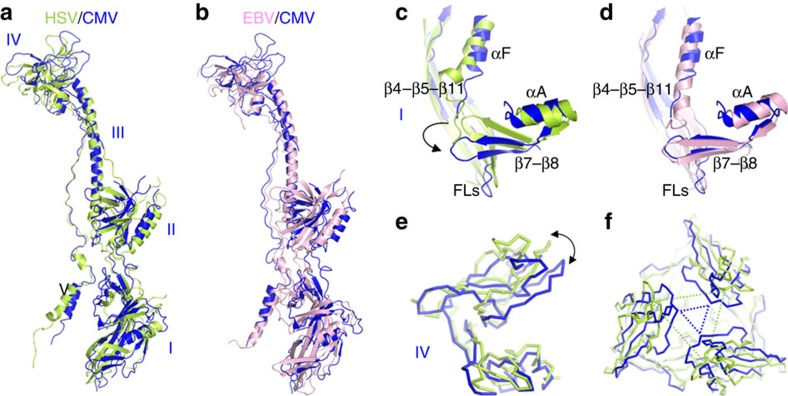
Comparison of HCMV and other herpesvirus gBs. HCMV gB is shown in blue, HSV-1 gB (PDB ID 2GUM^11^) in green and EBV gB (PDB ID 3FVC^24^) in pink. (**a**,**b**) Superimposition of HCMV gB and HSV gB (**a**) or EBV gB (**b**) based on alignment of the central helix (domain III). (**c**,**d**) Superimposition of domain I of HCMV and HSV gB (**c**) or EBV gB (**d**) shows that β-sheets 7 and 8, and the C-terminal alpha helix occupy positions more similar to EBV gB than HSV gB. The β-sheets 4, 5 and 11 contributing to the fusion loops are shown in the background. (**e**) Side view of a superimposition of domain IV of HCMV and HSV gB based on the positionally conserved β-sheets 33–35 at the base. (**f**) Top view of (**e**) showing the smaller pore in the HCMV gB trimer compared with HSV gB. The location of Glu610 in HCMV gB and its homologous Glu631 in HSV gB are indicated by triangles in blue and green, respectively. All superimpositions were carried out using LSQKAB in CCP4.

The sequence of HCMV gB shares roughly 30% identity with both HSV and EBV gBs. Taken individually, each of the HCMV gB structural domains superpose well with their counterparts in HSV and EBV (Supplementary Fig. 3). RMSD values range from 0.85 Å for the central helix to 2.96 Å for domain I (Supplementary Table 1) with most local differences occurring in loops. Domain II also very closely overlaps with the crystal structure of the isolated bacterially expressed domain II bound to SM5 Fab^37^, with an RMSD of 0.75 Å. However, when the three trimers are superimposed by aligning their highly conserved central helix, significant twists become apparent in the relative location of domains I, II and IV (Fig. 3a,b) with the HCMV gB structure occupying an intermediate position between HSV and EBV gBs. Also in the context of the trimer, within domain I, the β7–β8 loop of protomer A sits adjacent to β4, β5 and β11 of protomer B (that contribute to the fusion loops) and the αF helix of protomer C (Fig. 3c,d). This region differs between HSV and EBV gBs, with the β7-β8 loop occupying different positions to accommodate the exiting C-terminal tail^24^. This loop aligns better to the corresponding one from EBV gB (Fig. 3d), suggesting that the HCMV gB C-terminal tail, not present in the crystallized molecule, may follow a path similar to that of EBV gB.

Larger differences are seen in domain IV (crown) of gB, which is composed predominantly of β-sheets with a fold unique to herpesvirus gBs^11^. In a superimposition of the domain IV of HCMV and HSV gB based on β-strands β33–35, whose location is conserved in the two molecules, an inward rotation of the rest of the domain (β1 and β26–31) becomes apparent (Fig. 3e). This leads to formation of a smaller pore along the three-fold axis for HCMV gB compared with HSV gB (Fig. 3f). This domain is largely disordered in EBV gB, suggesting conformational flexibility of this region.

### gB glycosylation sites and surface charge distribution

HCMV gB has a larger number of *N*-linked glycosylation sites than the other herpesvirus gB proteins, with 18 predicted in the primary sequence of the wild-type (WT) ectodomain (Fig. 1a) compared with five and nine for HSV and EBV gBs, respectively. All the glycosylation sites are conserved across HCMV strains with the only differences occurring in the proximity of the furin cleavage site and in the flexible N terminus. Electron density was observed for glycosylation of 10 of the potential 16 sites in the ΔNgB-glyco crystal structure, with the remaining six sites occurring in disordered regions of the structure. The surface of gB between domain I and II (AD-4 and AD-5, respectively) is particularly heavily *N*-glycosylated with seven glycosylation sites between them (that is, positions 281, 286, 302 and 341 in domain I and 383, 405 and 417 in domain II). These domains have been implicated in interaction with gH/gL and the cell membrane in HSV gB^13^,^41^, and heavy glycosylation in their proximity may have evolved to mask these functional sites from the host antibody response.

The overall surface charge distribution on HCMV gB is distinct from that seen with HSV or EBV gB^24^ (Supplementary Fig. 4). This difference is most apparent in the crown, which has a positive patch unique to HCMV gB contributed by residues Arg563 and Lys634, enclosing a negatively charged inner groove towards the trimeric axis. At the base of the molecule, the fusion loops enclose a highly positively charged central groove, reminiscent of EBV gB. It has been proposed that the positive charge in this region may play a role in the interaction of gB with negatively charged phospholipid heads when the fusion loops insert into the membrane^24^.

### gB/1G2 interface

**Figure 4:**
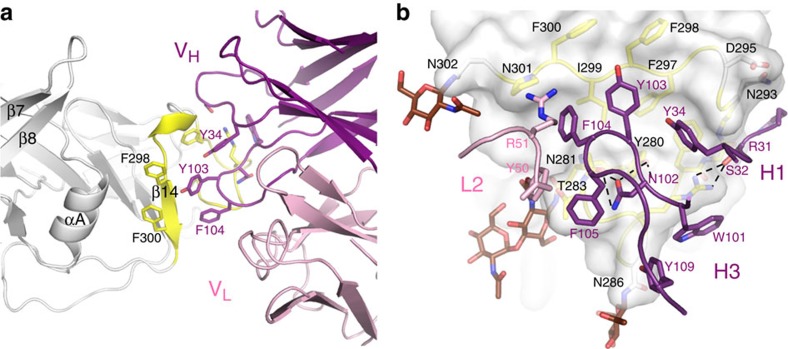
The gB/1G2 interface. (**a**) Overall view of the gB/1G2 interface. gB domain I is shown in white with residues contacted by 1G2 in yellow. The most important residues involved in the interaction are shown in stick representation. The view is rotated 180° from the image in Fig. 2c for better clarity. (**b**) Details of the gB/1G2 interaction with gB surface and key residues in stick representation. Only portions of HCDR1, HCDR3 and LCDR2 of 1G2 are shown. Hydrogen bonds between gB and the Fab are shown as dashed lines. Residue numbers for 1G2 heavy and light chains are shown in purple and pink, respectively. gB residues not forming direct contacts with 1G2 are shown in white. Glycosylated Asn residues at positions 281, 286 and 302 surrounding the epitope are shown with the linked NAGs in brown.

1G2 binds to a region of domain I (AD-5) encompassing a hydrophobic patch distinct from the fusion loops. Notably, 1G2 contacts gB mainly using heavy chain CDR1 and -3 (HCDR1 and -3), and establishes only limited interactions through the light chain CDR-2 (LCDR2; Fig. 4a and Supplementary Table 2). Over 1,350 Å^2^ of surface area is buried in the complex, which is well within the range of 1,200–2,000 Å^2^ observed for other antigen–antibody complexes^42^. The large buried surface area and surface complementarity of the gB/1G2 interface (*S*_c_=0.637; 0.64–0.68 is the range for most antigen-ntibody complexes) are consistent with the tight binding affinity of the complex with a *K*_D_ of 0.17 nM as measured by surface plasmon resonance (SPR) (Table 2).

Both hydrophobic and polar contacts contribute to the binding of gB to 1G2 (Fig. 4b and Supplementary Table 2). The aromatic side chains of HCDR3, Tyr103, Phe104 and Phe105 along with Asn102 and HCDR1's Tyr34 fill a hydrophobic cleft on the surface of gB consisting of residues Tyr280 and the stretch between Phe290 and Phe300. In addition, the binding of 1G2 seemingly leads to a kink in the gB backbone allowing the side chain of Arg285 to stack against the indole ring of HCDR3 Trp101 (Fig. 4b). HCDR1 residues Arg31 and Ser32 make polar contacts with the gB main chain atoms between Phe290 and Ala294. In addition, Ser32 forms polar contacts with the side chain of gB Arg285. The 1G2 light chain appears to have a more limited role in the interaction with gB. The side chain–OH groups of LCDR2 Tyr50 and gB Thr283 are 3.5 Å apart and may form a water-mediated hydrogen bond. Furthermore, together with LCRD2 Arg51, Tyr50 contributes to the positioning of HCDR3 (Fig. 4b).

### Molecular determinants of the gB/1G2 interaction

To support the structural findings and gain additional insights into the gB/1G2 interaction, we first mutated gB residues that are in proximity or part of the binding interface and measured their effect on the interaction using SPR (Table 2). The Arg285Ala mutation completely abolishes binding to 1G2. This result is in agreement with the extensive contacts established by the side chain of Arg285 with 1G2 (Fig. 4b and Supplementary Table 2). A number of other gB residues at the interface with 1G2 also contribute to complex formation. Point mutations of Glu292 or of hydrophobic residues Phe290, Phe297 and Ile299 to Ala significantly compromise the kinetics of the interaction, with the double mutant ΔNgB-glyco Phe298Ala/Ile299Ala having almost no binding to 1G2 (Table 2).

Of note, single point mutations of Tyr280 or Asn284 to Ala were previously shown to abrogate gB binding to 1G2 (ref. 36). Also, mutating Tyr280 affected the binding of gB to SM10 and 2C2. The structure reveals that in addition to contacting 1G2 (Fig. 4b), Tyr280 forms hydrogen bonds with both exposed and buried residues of gB, suggesting that it plays a role in preserving the structural integrity of the epitope. Consistent with this role, the Tyr280Ala mutation significantly reduced binding of 1G2 to ΔNgB-glyco as measured by SPR (Table 2 and Supplementary Table 3). While the Asn284Ala mutation was previously shown to reduce binding to 1G2 in an enzyme-linked immunosorbent assay (ELISA)^36^, the effect on the interaction was more subtle in our SPR experiments, and similar to three other mutations (Asn281Ala, Thr283Ala and Phe298Ala). Finally, Asn293 and Asp295, two residues that are critical for binding of gB to two other AD-5 specific mAbs SM10 and 2C2 (ref. 36), are in close proximity of the 1G2 epitope (Fig. 4b). However, these residues do not form direct contacts with 1G2 and when mutated to Ala, have minimal effect on 1G2 binding to gB (Table 2).

In contrast, mutation of Asn286 to Ala increased the overall gB/1G2 affinity (Table 2 and Supplementary Table 3). Interestingly, a recombinant virus carrying the same mutation was found to be more sensitive to neutralization by 1G2 (ref. 36). This residue falls within a canonical glycosylation site (^286^NAS^288^) and is exposed and glycosylated in the gB/1G2 structure (Fig. 4b). The absence of this glycan is expected to increase accessibility of 1G2 to its epitope resulting in tighter binding and, subsequently, higher neutralization potency.

To identify residues in 1G2 that are critical for gB binding and neutralization, we generated a panel of nine 1G2 variants carrying point mutations of CDR residues located in the gB/1G2 interface (Fig. 4b and Supplementary Table 2). Mutation of 1G2 HCDR3 Asn102, Tyr103 and Phe104 had the most profound effect on binding (Table 2). Substituting any of these residues with Ala increased the K_D_ by an order of magnitude, mainly due to a faster off rate. In particular, a Tyr103Ala/Phe104Ala double mutant was severely compromised in its ability to bind ΔNgB-glyco with only very weak binding detected. By contrast, point mutation of residues further away from the interface (HCDR3 Tyr109Ala, HCDR1 Arg31Ser, LCDR2 Tyr50Ala or Arg51Ala) did not have any significant effect on the interaction. Surprisingly, the HCDR1 Ser32Ala mutation enhanced binding. It is possible that removing the polar side chain may allow the backbone carbonyl of residue 32 to interact with Arg285 without interference from the side chain hydroxyl of a Ser.

We next tested the 1G2 mutants in a virus neutralization assay. ARPE-19 epithelial cells were infected with HCMV (TB40/E strain) in the presence of WT 1G2 or its various mutants (Table 2). In line with the SPR data, the mutants of 1G2 that were compromised in their ability to bind to gB had higher EC_50_ values compared with WT 1G2 (Table 2). It is likely that avidity effects, due to the bivalent nature of antibody binding, account for the moderate increase in EC_50_s (two to sixfold) observed for most of the mutations compared with the more pronounced effect observed on gB/1G2 binding affinities. However, the double mutant Tyr103Ala/Phe104Ala, which binds poorly to ΔNgB-glyco, had an EC_50_ value nearly 68-fold higher than WT 1G2 (6.79 μg ml^−1^ and 0.1 μg ml^−1^, respectively). In contrast, other mutations in HCDR1 (Arg31Ser and Ser32Ala), HCDR3 (Tyr109Ala) and LCDR2 (Tyr50Ala and Arg51Ala) did not alter the neutralizing capacity of 1G2 significantly. Overall, the results of the mutagenesis studies are in agreement with the structural data.

### Functional characterization of 1G2

To further functionally characterize 1G2, we tested its breadth of neutralization on a number of HCMV clinical and laboratory strains (Table 3). 1G2 was able to effectively neutralize entry of all 10 strains tested in both epithelial and fibroblast cells in a complement-independent manner with average EC_50_ values of 0.246 μg ml^−1^ (1.64 nM) or 0.311 μg ml^−1^ (2 nM), respectively. We also assessed potential differences between 1G2 IgG and Fab by investigating their neutralization properties against the HCMV clinical strain VR1814. As previously reported^36^, the Fab of 1G2 was as effective as the whole IgG in neutralizing the virus in epithelial cells, with an EC_50_ value of 0.15 μg ml^−1^ (3 nM) compared with 0.21 μg ml^−1^ (1.42 nM) for the IgG (Table 3).

To gain insights into the molecular basis for the broad neutralizing properties of 1G2, we aligned the sequences of several clinical and laboratory strains of gB (Supplementary Fig. 5) and polymorphisms in the structured parts of the ectodomain were mapped to the surface of HCMV gB (coloured in cyan in Fig. 6a). It is apparent that unlike the AD-1 epitope, no known polymorphisms map to the epitopes of 1G2 mAb in AD-5 or SM5 mAb in AD-4, further underscoring the broad neutralizing capacity of these mAbs. One polymorphism at position 287 (Ala or Thr) falls in the proximity of the 1G2 epitope, but in the structure, Ala287 is solvent exposed and does not form any contacts with the Fab (Fig. 4b), consistent with the similar neutralization potency of 1G2 on both variants (Table 3).

**Figure 5:**
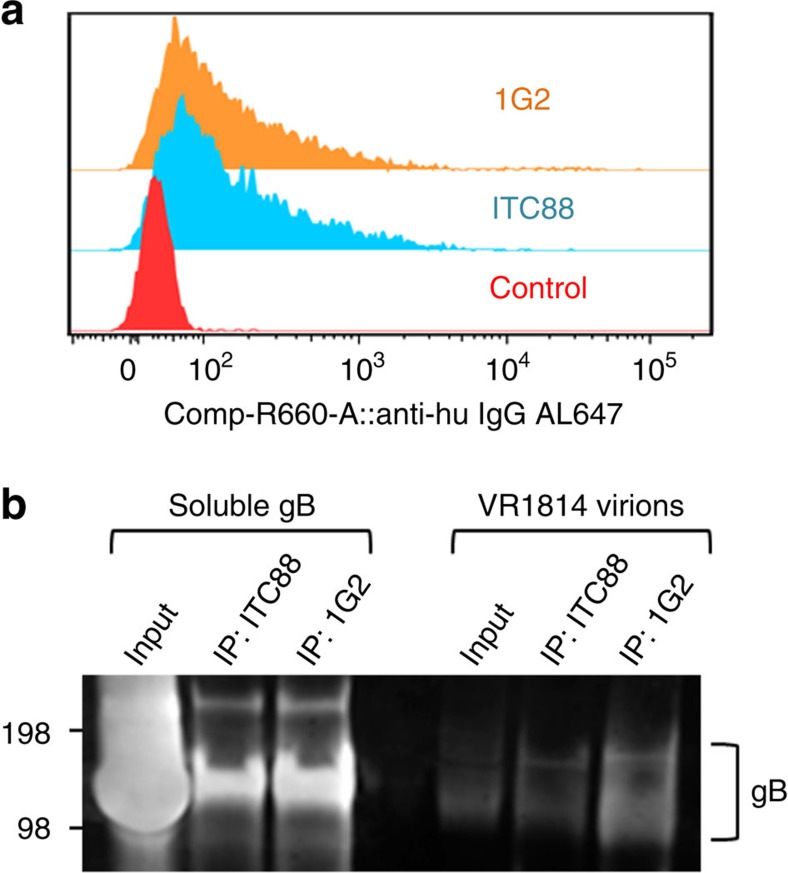
Binding of 1G2 to full-length gB on cell and viral surface. (**a**) FACS analysis of BHK cells electroporated with RNA encoding full-length gB and stained with monoclonal antibodies 1G2 and ITC88 24 h post electroporation. The gB staining histograms were gated on viable, dsRNA+ cells. (**b**) Western blot analysis of purified VR1814 virions immunoprecipitated with 1G2 or ITC88. Samples were resolved on SDS–polyacrylamide gel electrophoresis and detected with ITC88 mAb. The left panel shows immunoprecipitation of soluble gB-698glyco by both antibodies as control.

Finally, we tested whether 1G2 could bind to gB in the context of the membrane-anchored full-length protein. BHK cells were electroporated with RNA encoding full-length WT HCMV gB and the cell surface was stained with 1G2 or ITC88 (an antibody targeting the gB AD-2 epitope previously shown to bind cell surface-expressed and virion gB^43^). Both antibodies recognized gB expressed on the cell surface to a similar extent. Next, purified VR1814 virions were immunoprecipitated with 1G2 to determine if the epitope is accessible on the viral surface (Fig. 5b). ITC88 was used as a positive control. The 1G2 antibody immunoprecipitated gB from virions, confirming that the epitope is accessible on the viral surface. Together these results suggest that 1G2 binds to a functionally important site that is exposed and accessible to the antibody on both the cell and viral surfaces.

### Mapping known neutralizing epitopes on HCMV gB

**Figure 6:**
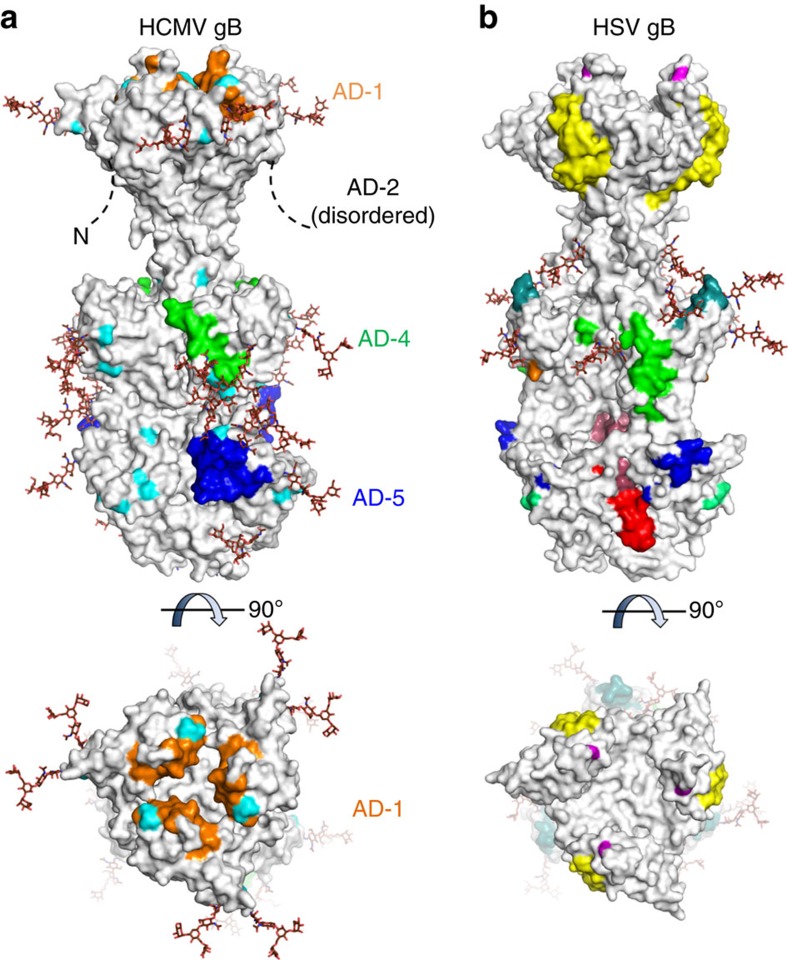
Neutralizing epitopes in HCMV gB. (**a**) Surface representation of HCMV gB in its postfusion conformation with known neutralizing epitopes in AD-1 (domain IV) in orange, AD-4 (domain II) in green and AD-5 (domain I) in blue. These epitopes are composed of residues Tyr280-Phe300 in AD-5 (mAb 1G2, this report), residues Ala360-Tyr364 and Ser377-Asp385 in AD-4 (mAb SM5 (ref. 37)) and residues Pro571-Val580 and His606-Phe619 in AD-1 (mAbs ITC48, ITC52, ITC63B and ITC63C^32^). The epitopes in AD-4 and AD-5 have been defined based on crystal structures while the epitope in AD-1 was identified through peptide mapping. Polymorphisms among clinical and laboratory strains of HCMV are shown in cyan. (**b**) Surface representation of HSV gB (PDB ID: 2GUM) with neutralizing epitopes defined through peptide mapping and single amino acid resistant mutant studies^11^,^31^,^64^. Residues recognized by mAbs H233 (Ala315 and Arg328), H126 (Tyr303), H1375 (Arg304), B4 (Glu305) and H1435 (His308) are shown in blue; mAb SS55 (Asp199 and Ala203) in light green; mAb H1781 (Pro454-Ser473) in teal; mAb H1838 (Ala390-Gly410) in green; mAb C226 (Asp419) in orange; mAb SS10 (Tyr640-Phe670) in yellow; mAbs B2 and B5 (Gly594) in magenta; mAb SS106 (Ser697-Ala725) in pink; mAb SS144 (Arg715-Ala725) in red. Simple (oligomannose) glycans shown as brown sticks were modelled into glycosylation sites in the primary sequence of HCMV and HSV gB using the Glyprot server.

Neutralizing epitopes in HCMV and HSV gB have been identified using peptide mapping, escape mutants and more recently by site-directed mutagenesis^11^,^32^,^33^,^35^,^36^,^38^. While antibodies to HCMV gB AD-1, AD-4 and AD-5 recognize conformational epitopes, AD-2 antibodies recognize linear sequences in the flexible N terminus of gB^32^. In a comparison of the known sites mapped on the respective postfusion gB structures (Fig. 6a,b), the footprint of 1G2 Fab overlaps with residues in HSV gB known to be critical for binding of several neutralizing antibodies (for example, H126, H1375, B4 and H1435)^11^. Likewise, the epitope for SM5, recently characterized in a crystal structure with an isolated AD-4 domain^37^, overlaps with that for H1838 in HSV gB. The known epitope in AD-1 is in a groove on the top of the crown and close to the trimer axis^32^. A point mutation conferring resistance to the B2 and B5 antibodies in HSV gB falls in a similar location. By contrast, an additional neutralizing epitope in HSV gB targeted by the SS10 antibody and mapped to the outer surface of the crown^31^ (Fig. 6b) has not been described for HCMV gB.

### Depletion of neutralizing antibodies in human sera by gB

To assess the contribution of antibodies targeting different HCMV envelope glycoproteins to neutralization, sera from five seropositive subjects were incubated with purified Pentamer, gH/gL^44^, or postfusion gB (gB-698glyco) and the corresponding antibody-depleted sera were evaluated for neutralization of epithelial cell and fibroblast infection (Table 4). The neutralization experiments were performed in the presence of complement because virion gB raises for the most part complement-dependent neutralizing responses. The specificity and efficiency of depletion were confirmed with an ELISA (Supplementary Fig. 6). As previously reported, HCMV Pentamer was confirmed to be the main target of antibodies inhibiting HCMV infection of epithelial cells^45^. Notably, while immunization with gH/gL is capable of raising antibodies that inhibit HCMV infection in both fibroblasts and epithelial cells^44^, no reduction in sera neutralization of epithelial cell infection was noted on depletion with gH/gL, reflecting the superior potency of antibodies specific for the pentamer in neutralizing epithelial cell infection^34^,^46^. Similarly, no observable difference in neutralizing titer is noted following depletion with purified postfusion gB when assayed on epithelial cells (Table 4), despite the exposure of the 1G2 epitope on virion gB (this study). By contrast, purified gB-698glyco was able to deplete a large fraction of the complement-dependent neutralizing activity of fibroblast infection by HCMV (Table 4). For three of the five subjects, depletion with gB brought neutralizing titres to below the detection limit, while the other two were reduced to nearly a third of the original titres. Interestingly, neither Pentamer nor gH/gL complexes showed significant neutralization depletion for fibroblast infection. Therefore, most of the epitopes recognized by gB-specific antibodies contained in sera from seropositive individuals and capable of neutralizing fibroblast infection in presence of complement are exposed on the postfusion gB molecule.

## Discussion

HCMV is a medically relevant human pathogen and a target for therapeutic intervention. HCMV gB is a key target for antibody development and remains a likely component of any future HCMV vaccine^17^. Here we have reported the crystal structure of an engineered homogeneous postfusion HCMV gB ectodomain trimer bound to a Fab derived from the neutralizing human monoclonal antibody 1G2. This represents the first atomic structure of an HCMV envelope glycoprotein.

1G2, SM10 and 2C2 antibodies were isolated from B-cell repertoire analysis from three seropositive subjects and mutagenesis studies suggested that they bind to the AD-5 site in gB^33^. Our EM analysis and the crystal structure of the complex confirmed the location of the epitope and revealed the atomic details of this interaction. 1G2 contacts gB mainly with the V_H_ domain burying a large hydrophobic surface on gB. The almost exclusive use of the heavy chain for binding is unusual^47^ and reminiscent of the influenza virus broadly neutralizing antibody CR6261 that, similar to 1G2, binds to a hydrophobic surface on the HA molecule^48^. The interaction is characterized by 1G2 HCDR3, rich in aromatic amino acids, and HCDR1 protruding into a hydrophobic cleft within AD-5. Our mutagenesis data confirmed the critical role of several of these aromatic residues for tight binding to gB and virus neutralization.

It is interesting to note that among all the isolated neutralizing mAbs targeting gB AD-5, 1G2 has the shortest HCDR3 (12 residues versus 17 for SM10 and 24 for 2C2) (refs 33, 36). In addition, sequence comparisons reveal that not all of the AD-5 antibodies have hydrophobic CDRs, suggesting that presence of hydrophobic CDRs is not an absolute requirement for binding to the AD-5 site^33^. Of note, 1G2, SM10 and 2C2 belong to different germlines suggesting that there is no specific genetic requirement to elicit antibodies binding to this epitope.

The structure of postfusion gB reveals the location and structure of epitopes recognized by other neutralizing human mAbs, and our structural analysis demonstrates that most of these epitopes are common to both HCMV and HSV gBs. This observation suggests a conserved functional role of these sites across herpesvirus gBs. A recent report described the structure of an HCMV AD-4 only molecule bound to the SM5 Fab^37^. The postfusion gB trimer structure confirms that this epitope is exposed and has a very similar structure in the context of the entire gB ectodomain. AD-1, on the crown, was identified using peptides. Interestingly, when mapped on the postfusion gB structure, this site seems to point towards the three-fold axis. Therefore the epitope is likely not accessible to antibodies in the postfusion conformation. This observation points to a potential limitation of the postfusion gB molecule as a subunit vaccine antigen in eliciting neutralizing antibodies binding to this site in AD-1.

Mouse immunization studies with different postfusion HCMV gB trimer preparations were shown to raise almost exclusively complement-dependent neutralizing antibodies^44^,^49^. However, the structure of postfusion gB reveals that sites capable of binding complement-independent neutralizing antibodies are present in this form of the gB molecule suggesting that other epitopes are dominant. Consistent with this hypothesis, the structure also showed that both AD-4 and AD-5 are surrounded by glycosylation sites, which may limit epitope exposure, and the neutralizing site in AD-2 may be only partially accessible to antibody binding. The situation seems similar to the case of natural infection where only a small percentage of the antibodies targeting gB are neutralizing^33^,^50^. We speculate that, similar to other viruses^51^, the majority of the fusion proteins on the viral surface are in the postfusion conformation providing an escape mechanism. Therefore, antigen design strategies further modifying the postfusion gB trimer to focus the immune response towards neutralizing sites (for example, AD-4 and AD-5) and dampening the non-neutralizing sites may result in an improved vaccine antigen. For instance, selective mutation of glycosylation sites surrounding the 1G2 epitope may increase its exposure and elicit more neutralizing antibodies binding to this epitope.

In summary, the structure of the postfusion HCMV gB trimer reveals structural and antigenic similarities with other herpesvirus gB likely due to the conserved functional role of these molecules. The structural and antigenic analyses show that the postfusion trimer presents complement-independent neutralizing sites. Thus modifications on the gB trimer surface that decrease exposure of non-neutralizing sites may result in an improved vaccine. Conversely, potent HCMV neutralizing antibodies, like 1G2, are attractive candidates for treatment of HCMV infection. Individual mAbs, or combinations of neutralizing antibodies targeting the different domains within gB and/or the HCMV gH/gL-based complexes may provide a suitable strategy.

## Methods

### Generation of HCMV glycoprotein and Fab constructs

Merlin strain HCMV gB codon-optimized for expression in mammalian cells, with an optimal Kozak sequence immediately 5' to the gene, was synthesized by GeneArt (Regensburg, Germany) and subcloned into the *Sal*I and *Xba*I restriction sites of the eukaryotic expression vector pCMVKm2 (ref. 52). The gB gene was truncated at amino acid 698 to remove the transmembrane domain and cytoplasmic tail and fused to a 6-histidine tag for purification purposes. Point mutations of Ile156His, His157Arg, Trp240Arg, Cys246Ser, Arg457Ser, Arg460Ser, were introduced by site-directed mutagenesis using a QuikChange II XL Site Directed Mutagenesis Kit (Agilent) resulting in gB-698. Addition of the mutations Trp240Asn and Tyr242Thr to gB-698 or deletion of residues 26–87 together with the Trp240Asn and Tyr242Thr mutations in gB-698 generated gB-698glyco and ΔNgB-glyco, respectively. Recombinant 1G2 IgG and Fab were cloned based on the published heavy and light chain sequences^33^. The recombinant IgG was cloned into an IgG1 backbone. The Fab was expressed with a cleavable C-terminal Strep tag II on the heavy chain.

### Expression and purification of gB, 1G2 and gB/1G2 complexes

1G2 Fab was transiently expressed in 293EBNA cells (ATCC, Manassas, VA), gB-698glyco was stably expressed in CHO lec^−^ cells whereas ΔNgB-glyco was transiently expressed in HEK293S GnTI^−^ cells (ATCC, Manassas, VA). For purification of all the gB constructs, expression media were concentrated 10-fold and buffer exchanged into 25 mM Tris pH 7.5, 300 mM NaCl using a tangential flow filtration system (Millipore). Imidazole to a final concentration of 10 mM was added to sample before loading onto a Ni-NTA Superflow cartridge (Qiagen) using the Akta Explorer 100 Air system (GE Lifesciences). His-tagged gB was eluted using a continuous gradient of 10–500 mM imidazole with the protein typically eluting around 200 mM. Fractions dialysed into 25 mM Tris pH 7.5, 150 mM NaCl were further purified using size exclusion chromatography on a Superose 6 column (GE Lifesciences). The proteins were concentrated to final concentrations of 7 mg ml^−1^for gB-698glyco and 3.5 mg ml^−1^ for ΔNgB-glyco. Strep-tagged 1G2 Fab was affinity purified using a *Strep*-Tactin Superflow Plus column (Qiagen) and concentrated to 5.3 mg ml^−1^.

For deglycosylation experiments, Ni-purified gB ectodomain constructs were incubated at room temperature (RT) for 20 min with Endo Hf (New England Biolabs) according to manufacturer's instructions. The protein was subsequently purified by SEC as described above and concentrated to 3.7 mg ml^−1^ (gB-698glyco) or 1.74 mg ml^−1^ (ΔNgB-glyco).

### EM for gB and gB/1G2 Fab complexes

For EM analysis, gB-698glyco was incubated with 1G2 Fab at a 1:1.5 molar ratio on ice for 1 h and resolved over a Superose 6 PC 3.2/30 column connected to an Akta Micro system (GE Lifesciences). For visualization, 5 μl of sample was incubated on a freshly discharged continuous carbon 400-mesh copper grid (Electron Microscopy Sciences) for 30 sec. Following incubation, the grid was moved through 5 × 75 μl droplets of a 2% (w/v) uranyl formate solution (SPI Supplies). Excess stain solution was blotted away and the grid was air-dried. EM images were collected on a Tecnai-12 Spirit (FEI) equipped with a LaB6 filament operated at 120 keV under low dose conditions using a 4 k × 4 k CCD camera (Gatan Inc.) at a nominal magnification of × 49,000 (2.15 Å per pixel). Particles were isolated from individual micrographs using the e2boxer.py procedures embedded into EMAN2 (ref. 53) and subjected to iterative two-dimensional reference-free image analysis using multivariate statistical analysis followed by multi-reference alignment in IMAGIC5 (ref. 54). To obtain a gB/1G2 three-dimensional reconstruction, ∼10,000 particles were refined against an initial model of HSV gB (PDB ID 2GUM) low pass filtered to a resolution of 60 Å using the EMAN2/Sparx software^53^,^55^ and fitted in Chimera^56^.

### Crystallization of gB/1G2 Fab complexes

All crystallization trials were carried out at 20 °C. In initial crystallization experiments, gB-698glyco was mixed with 1G2 Fab at a molar ratio of 1:1.2, and 1,000:1 w/w of Subtilisin E (Hampton Research) and directly screened using sparse matrix crystallization screens. Crystallization screens were set-up using a Mosquito Crystal liquid handler (TTP Labtech) in 96-well hanging drop format and imaged using a RockImager robot (Formulatrix Inc). Initial crystals appeared overnight in a drop containing 0.1 μl protein and 0.1 μl reservoir solution of 0.1 M MMT buffer pH 9.0, 25% PEG-1500 (Wizard IV crystallization screen, Emerald Biosystems). Final crystals were obtained in 15-well screw cap hanging drop plates (Qiagen Inc) by microseeding into 0.1 M MMT pH 8.4, 12% PEG-1500, 5% glycerol after overnight equilibration. Screw caps with crystals were transferred to a well containing 0.1 M MMT pH 8.4, 25% PEG1500, 20% glycerol for dehydration overnight, and crystals were directly flash-frozen in liquid nitrogen. Crystals of deglycosylated ΔNgB-glyco/1G2 Fab were obtained under similar conditions with no protease treatment, and used as seeds for obtaining single crystals in a drop containing a 1:1 volume ratio of protein complex to 40 mM MMT pH 9, 8% PEG8K and 0.1 M NaCl and 20% glycerol. Crystals were directly flash frozen in liquid nitrogen for data collection.

### Structure determination of ΔNgB-glyco/1G2 Fab complex

Data collection was carried out with a 50% attenuated beam at the PXII beamline at the Swiss Light Source (SLS, Switzerland). Data were scaled and processed using XDS autoPROC (Globalphasing Inc) to 3.6 Å resolution for subsequent analysis and structure determination. Matthew's coefficient analysis within the CCP4 suite^57^ predicted a single monomer per asymmetric unit with 67% solvent content. Molecular replacement (MR) was carried out using Phaser (CCP4 suite)^58^ with the HSV gB ectodomain structure as search model (PDB ID: 2GUM). A single solution with gB along the three-fold axis was obtained with an *R*_work_/*R*_free_ of 0.55/0.56. Initial electron density was clearest for the central helix while densities in structural domains I and IV were weakest. The initial gB model was further improved by iterative cycles of model building in Coot, model refinement using Sculpt^59^ within Phenix^60^ and electron density modification with DM (CCP4 suite). Cycles of model refinement with Phenix^61^ using only TLS parameters, *xyz* coordinates options combined with kicked electron density maps and model building in Coot^62^ were performed until the *R*_work_ and *R*_free_ reached 0.26 and 0.31, respectively.

For placing the Fab, a carbon alpha trace for HCDR3 was first built into a well-defined electron density at the complex interface. Then the V_H_V_L_ chains of the Fab could be located by molecular replacement with Phaser. No clear solution could be identified for the C_H_ and C_L_ domains of the Fab. After several cycles of model building and refinement the final *R*_work_ and *R*_free_ values decreased to 0.21 and 0.26, respectively (Table 1). While the crystal packing allows enough space for the constant region of the Fab, the electron density is mostly discontinuous suggesting that this region is flexibly linked to the variable region and therefore not visible because it likely adopts different orientations in the crystals. Structure validation was carried out using Molprobity^63^. The coordinates for the structure have been deposited with the RCSB Protein Data Bank under PDB ID 5C6T.

### SPR experiments for gB/1G2 binding

SPR single cycle kinetic experiments were carried out using a Biacore T100 instrument. Two adjacent channels on a CM5 sensor chip were immobilized with IgG binder using the Human Antibody Capture Kit (GE Healthcare BR100839) according to manufacturer's recommendations. HBS-EP buffer (GE healthcare BR100669) was used as diluent for both ligand and analyte samples. Ligand 1G2 IgG was captured on one channel (leaving the other channel as reference). For the 1G2 mutants, cell culture supernatant expressing the various mutants was flowed over the human IgG binder for the capture step. Capture levels were around 200 resonance units (RUs) for analysis of the ΔNgB-glyco mutants and roughly 40 RUs for analysis of the 1G2 mutants. For both experiments, ΔNgB-glyco at 0, 1.56, 3.125, 6.25, 12.5 and 25 nM were injected over the two channels at 50 μl min^−1^ for 120 s followed by 600 s of dissociation time. The single cycle kinetic curves were fitted with 1:1 binding stoichiometry for *k*_a_, *k*_d_ and *K*_D_.

### Determination of concentration of 1G2 mutants using SPR

To determine the concentration of 1G2 and its point mutants in cell culture supernatant, an SPR-based concentration analysis method was used. Briefly, a calibration curve was set-up using purified 1G2 at concentrations ranging from 10 μg ml^−1^ to 3.2 ng ml^−1^ in fivefold dilutions by capturing the antibody on a human IgG binder chip described above. A 1:100 dilution of cell culture supernatant from 293 Expi cells (Invitrogen Inc.) transfected with WT or point mutants of 1G2 was then flowed over the chip and the resulting capture level recorded. The final concentration of the antibodies was determined by the concentration analysis using calibration program within the Biacore T200 evaluation software.

### Surface staining of full-length gB by flow cytometry

BHK cells were electroporated with 0.1 μg of FL-gB RNA along with 4.2 μg of mouse thymus RNA. Following overnight culture (16–18 h) the cells were detached using Trypsin-EDTA (Invitrogen Inc.). Cells were initially stained for viability using a LIVE/DEAD Fixable Yellow Dead Cell Stain Kit (Life Technologies) and were then stained for surface expression of gB with either ITC88 (1 μg per sample), 1G2 (1 μg per sample) or none (as control), followed by goat anti-human IgG (H+L) Alexa Fluor 647 (0.25 μg per sample; Cat no. A-21445, Life Technologies). Cells were fixed and permeabilized, and then stained with an anti-dsRNA mAb (0.25 μg per sample; clone J2, Cat no. 10010500, English and Scientific Consulting Bt) that had been conjugated using a Zenon R-Phycoerythrin Mouse IgG2a Labeling Kit (Life Technologies). All samples were analysed by flow cytometry on an LSR II instrument (BD Biosciences).

### Virus neutralization assay with 1G2 mutants

1G2 WT or various point mutants were expressed in 293Expi cells in a 24-well format. Supernatant from all samples were verified for antibody expression 4 days post-transfection on a Coomassie-stained SDS–polyacrylamide gel electrophoresis gel. Supernatant samples were serially diluted in twofold steps (two replicates per dilution), mixed with an equal volume of HCMV virus (concentration of 200–250 infected cells/counting field) in media+10% guinea pig complement (Cedarlane Labs, Burlington, NC, USA) and incubated for 2 h at 37 °C/5% CO_2_. These serum/virus samples were added to ARPE-19 cells prepared in 96-well half-area cell culture plates (Corning Inc., Corning, NY, USA) plated at a density of 6.25 × 10^4^ per well 1 day before infection. The infected monolayers were incubated for 48 h (±8 h) at 37 °C/5% CO_2_, fixed with 10% buffered formalin (EMD Chemicals Inc., Gibbstown, NJ, USA) for 1 h, washed three times with wash buffer (PBS/0.05% Tween-20) and blocked with PBS/2.5% FBS, 0.5% saponin and 0.1% sodium azide for 1 h at RT. Following three washes, plates were tapped dry and incubated in a 25 °C humid incubator for 1 h. The plates were then incubated for 1 h at RT with anti-HCMV IE1 antibody derived from hybridoma L14. Plates were washed three times and incubated for 1 h with anti-mouse IgG conjugated with Alexa Fluor 488, and then washed three times with PBS/0.05% Tween-20. The fluorescent cells were counted using an Immunospot S5 UV Analyzer (Cellular Technology Limited, Shaker Heights, OH, USA), and the 50% neutralization titer, defined as the reciprocal of the serum dilution yielding 50% reduction in the infected cell count relative to control, was calculated by linear regression interpolation between the two dilutions with wells yielding average infected cell counts above and below the 50% value.

### Human sera depletion assay

Sera from five human subjects seropositive for CMV antibodies were obtained from SeraCare Life Sciences (Milford, MA, USA). Serum samples were heat inactivated at 56 °C for 30 min and subsequently incubated for 1 h at RT with and without purified soluble gB-698glyco, gH/gL or pentamer at a concentration of 40 μg ml^−1^. These depleted and non-depleted samples were then assayed in the neutralization assay as described above. To determine the specificity and efficiency of depletion, sera incubated with soluble gB-698glyco, gH/gL or Pentamer as described above were assayed in an ELISA with the respective proteins coated onto plates. Sera incubated with an unrelated protein (‘mock') were used as a control.

### Neutralization assay with multiple strains of HCMV

HCMV virus from various strains was pre-incubated with 10-fold serial dilutions of 1G2 antibody (from 100 to 0.0001 μg ml^−1^) for 1 h before infection. ARPE-19 or NHDF cells (ATCC, Manassas VA) seeded at 10,000 per well were infected in a 96-well plate with the virus antibody complex for 3 h in a serum free medium, followed by a wash with complete medium. Fourty-eight hours post infection, cells were fixed with 4% paraformaldehyde and permeabilized with 0.1% Triton-X100. Mouse anti-IE antibody 6F8.2 (Chemicon) was diluted 1:2,000 and incubated on the cells for 1 h at RT following which cells were washed three times in 1 × PBS. Anti-mouse AF594 (1:3,000) and DAPI (1:5,000) were diluted in 1 × PBS and incubated on cells for 1 h at RT. Cells were washed three times in 1PBS and the number of IE1/2 positive cells were counted using a Cellomics Arrayscan.

### gB immunoprecipitation

Soluble gB ectodomain or an equal amount of cushion-purified VR1814 virions (500 μg per reaction) were lysed in 1% NP40 and incubated with 10 μg of 1G2 or ITC88 antibodies for 16 h at 4 °C. Complexes were immunoprecipitated using Dynabeads Protein A kit from Life technologies (Cat. no. 10006D) according to the manufacturer's protocol. The complexes were washed four times in lysis buffer. Samples were boiled for 5 min at 96 °C before immunoblotting with ITC88. The uncropped western blot image is shown in Supplementary Fig. 7.

## Additional information

**Structural Data.** Structural data for the HCMV gB-1G2 complex were deposited in the RCSB Protein Data Bank under PDB ID 5C6T.

**How to cite this article:** Chandramouli, S. *et al*. Structure of HCMV glycoprotein B in the postfusion conformation bound to a neutralizing human antibody. *Nat. Commun.* 6:8176 doi: 10.1038/ncomms9176 (2015).

## Supplementary Material

Supplementary Information Supplementary Figures 1-7 and Supplementary Tables 1-3

## Figures and Tables

**Table 1:** X-ray data collection and refinement statistics.

	*Δ**NgB-glyco+1G2 Fab***
*Data collection*	
Space group	P2_1_3
Cell dimensions	
*a*, *b*, *c* (Å)	176.49, 176.49, 176.49
α, β, γ (°)	90.0, 90.0, 90.0
Resolution (Å)	50–3.55 (3.74–3.55)*
No. of reflections	1,56,458 (22,493)
No. of unique reflections	22,423 (3,233)
*R*_merge_	0.104 (1.061)
*I*/*σI*	16.4 (2.1)
Completeness (%)	100 (100)
Redundancy	7.0 (7.0)
	
*Refinement*	
Resolution (Å)	19.983–3.6
No. reflections	21,341
*R*_work_/*R*_free_	0.212/0.260
No. atoms	
Protein	6,270
Ligand/ion	141
Water	—
B-factors	
Protein	116
Ligand/ion	180
Water	—
R.m.s. deviations	
Bond lengths (Å)	0.005
Bond angles (°)	1.147
Ramachandran plot†	
Favoured	85.8%
Allowed	11.5%
Outliers	2.7%

R.m.s. deviation, root-mean-square deviation.

^*^Values in parentheses are for highest-resolution shell.

^†^Measured using Molprobity.

**Table 2:** Molecular determinants of gB/1G2 binding.

**Sample**	***k***_**a**_ **(× 10**^5 ^**M**^−1^ **s**^−1^)	***k***_**d**_ **(× 10**^−5^ ** s**^−1^)	***K*****D** **(nM)**	**Fold difference from WT (KD)**	**EC50 (μg ml**^−1^)
*gB mutants*					
ΔNgB-glyco	3.70	6.36	0.17	1.00	NA
Y280A	0.07	19.50	29.30	170.35	NA
N281A	2.18	4.57	0.21	1.22	NA
T283A	3.10	6.99	0.23	1.31	NA
N284A	3.20	7.66	0.24	1.41	NA
R285A	—*	—	—	—	NA
N286A	7.24	4.89	0.07	0.39	NA
NRN-AAA†	—	—	—	—	NA
F290A	0.66	24.90	3.79	22.03	NA
E292A	1.32	10.80	0.82	4.75	NA
N293A/D295A	2.40	8.43	0.35	2.04	NA
YND-AAA‡	0.21	39.17	18.72	108.84	NA
F297A	0.19	26.70	13.70	79.65	NA
F298A	2.14	5.59	0.26	1.52	NA
I299A	1.55	9.00	0.58	3.37	NA
F298A/I299A	—	—	—	—	NA
*1G2*					
WT	2.95	3.75	0.13	1.00	0.10
*1G2 heavy chain*					
R31S	2.73	6.07	0.22	1.76	0.05
S32A	3.96	1.06	0.03	0.21	0.09
N102A	3.06	52.60	1.71	13.46	0.67
Y103A	3.25	76.60	2.36	18.58	0.31
F104A	2.94	45.00	1.53	12.05	0.20
Y103A/F104A§	—	—	—	—	6.79
Y109A	3.24	7.67	0.24	1.87	0.10
*1G2 light chain*					
Y50A	3.57	11.80	0.33	2.60	0.13
R51A	3.27	5.11	0.16	1.23	0.16

NA, not applicable.

Surface plasmon resonance data for various ΔNgB-glyco and 1G2 containing point mutations of residues located at the complex interface. Single cycle kinetics was performed on each pair. Further details are included in Supplementary Table 3. The neutralization potential of 1G2 and its mutants was assessed on ARPE-19 epithelial cells, with EC50 values reported here (far right column).

^*^=no measurable binding detected.

^†^NRN-AAA=N284A/R285A/N286A.

^‡^YND-AAA=Y280A/N293A/D295A.

^§^=very weak binding.

**Table 3:** Neutralization of various HCMV clinical isolates by 1G2.

**HCMV virus strain**	**Neutralization EC50s for 1G2 (μg ml**^−1^)		**Residue at position 287**
	**ARPE**	**NHDF**	
VR1814 (1G2 mAb)	0.213	0.077	Thr
VR1814 (1G2 Fab)	0.151	NA	Thr
TM-31354	0.230	0.411	Thr
8816	0.046	NA	Ala
8818	0.688	0.289	Thr
8819	0.485	0.850	Ala
8821	0.108	0.401	Thr
8824	0.019	0.043	Ala
8822	0.319	0.485	Ala
TR	0.248	0.303	Ala
TM-28175	NA	0.138	Ala
MP-MD-805	NA	0.115	Thr
MP-LW-1802	0.104	NA	Thr

HCMV, human cytomegalovirus; NA, not applicable; NHDF, normal human dermal fibroblasts.

EC50 values for various clinical HCMV isolates measured on epithelial cells (ARPE-19) and fibroblasts (NHDF). Infection of 50% cells was determined based on HCMV IE expression. NA, clinical isolate did not express IE on a particular cell type.

**Table 4:** Neutralization activity in human sera after antibody depletion with gB, gH/gL and pentamer.

**Cell type**	**Depleting protein**	**Human serum**				
		**1**	**2**	**3**	**4**	**5**
Fibroblasts	None	2,254	4,093	2,442	4,488	3,328
	gB-698glyco	100	100	768	1,266	100
	gH/gL	1,598	3,689	5,738	3,523	3,921
	Pentamer	1,607	1,363	2,240	3,761	4,569
Epithelial cells	None	4,126	11,407	⩾12,800	⩾12,800	4,867
	gB-698glyco	3,972	⩾12,800	⩾12,800	⩾12,800	5,086
	gH/gL	4,561	12,422	⩾12,800	⩾12,800	5,636
	Pentamer	720	1,104	1,408	1,708	1,464

gB, glycoprotein B; HCMV, human cytomegalovirus.

Sera from five convalescent seropositive human subjects were assessed for neutralization potential on fibroblasts (MRC-5 cells) following infection with HCMV Towne strain, or epithelial cells (ARPE-19 cells) following infection with HCMV TB40/E strain. The respective strains were chosen to achieve optimal infection of the epithelial and fibroblast cells. All neutralization assays were carried out in the presence of complement. Depletion with gB-698glyco, gH/gL or Pentamer was carried out by incubating the sera with the proteins for 1 h before the neutralization assay.
