# Supplementary material for: Structure of HCMV glycoprotein B in the postfusion conformation bound to a neutralizing human antibody
Source: Nat Commun. 2015 Sep 14;6:8176. doi: 10.1038/ncomms9176 (PMC4579600; doi:10.1038/ncomms9176)
Supplement: Supplementary Information — Supplementary Figures 1-7 and Supplementary Tables 1-3 [file ncomms9176-s1.pdf]

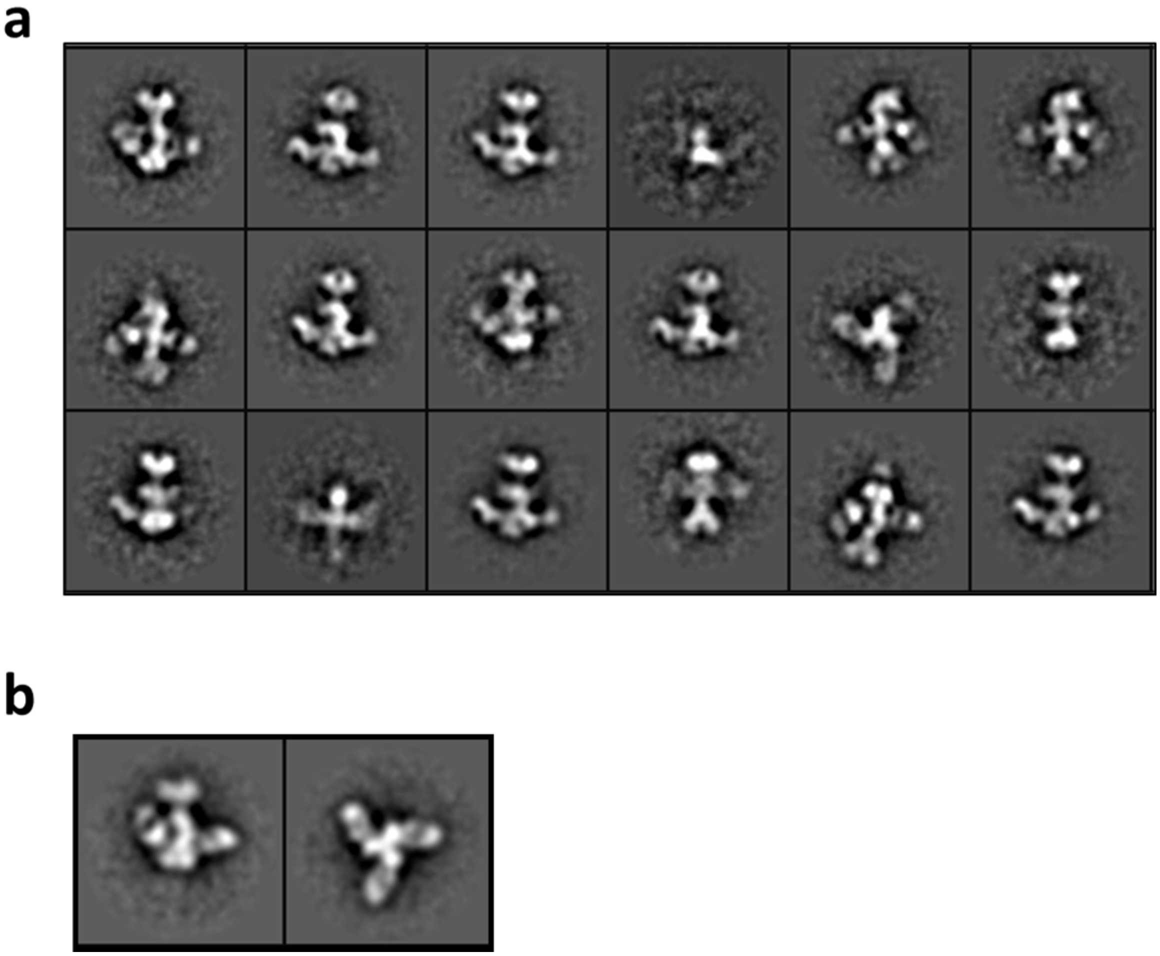

Supplementary Figure 1. Electron microscopy of gB-698glyco/1G2 Fab complex. a) Representative images of 2D class averages of gB-698glyc bound to 1G2 Fab. Top views of the complex were underrepresented compared to the side views. **b)** 2D class averages of redissolved crystals of Subtilisin E-treated gB-698glyco/1G2. Crystals of deglycosylated gB-698glyco/1G2 grown in the presence of a 1:1000 ratio of Subtilisin E were looped out of the crystal solution, washed three consecutive times in reservoir buffer (0.1M MMT pH 8.4, 12% PEG-1500, 5% glycerol) and dissolved in 25 mM Tris pH 7.5, 150 mM NaCl for visualization through EM as described in Materials and Methods. 2D class averages of side and top view of the complex are shown.

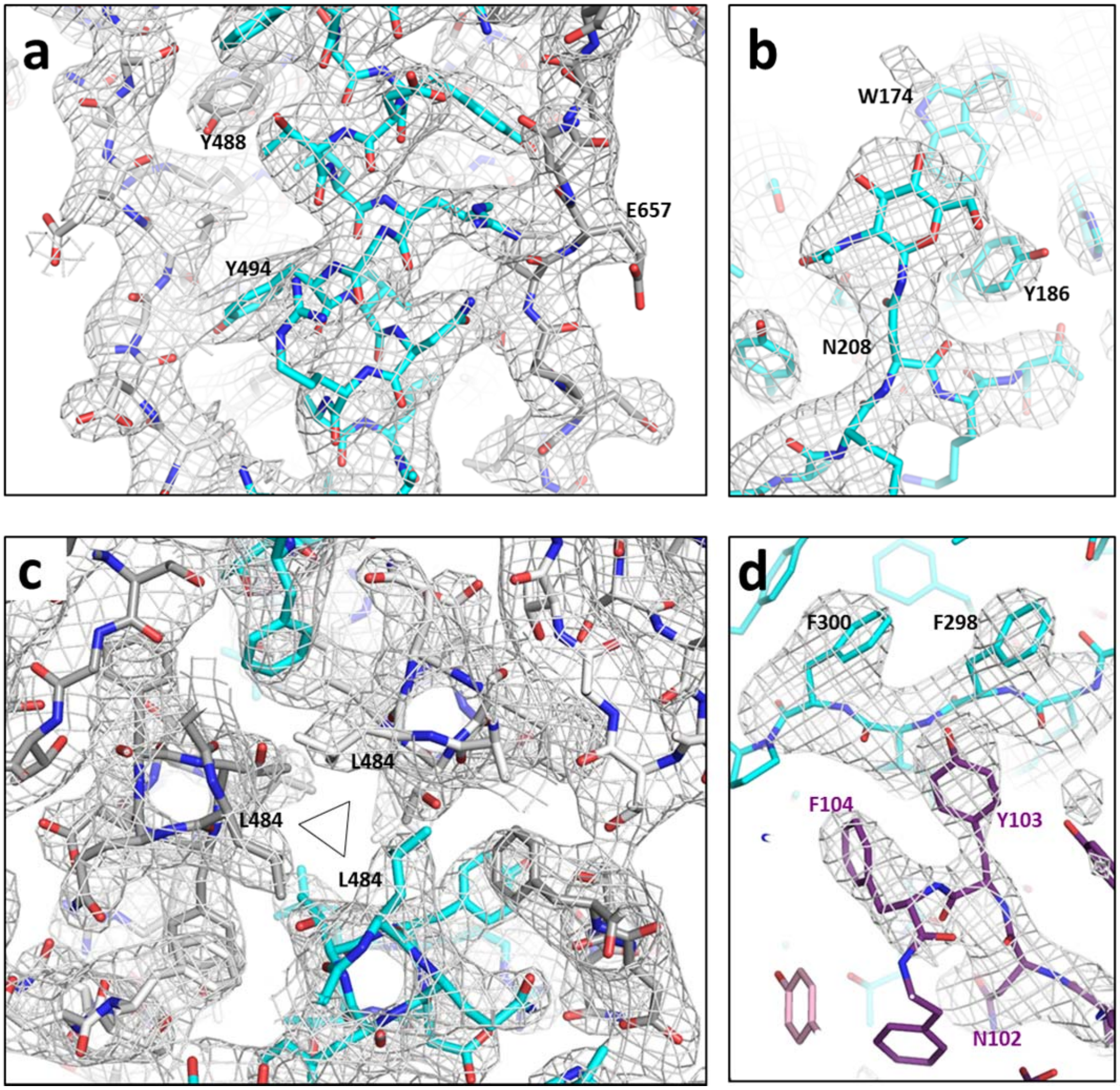
a
Y488
E657
Y494
b
W174
Y186
N208
c
L484
L484
L484
d
F300
F298
F104
Y103
N102

Contd.

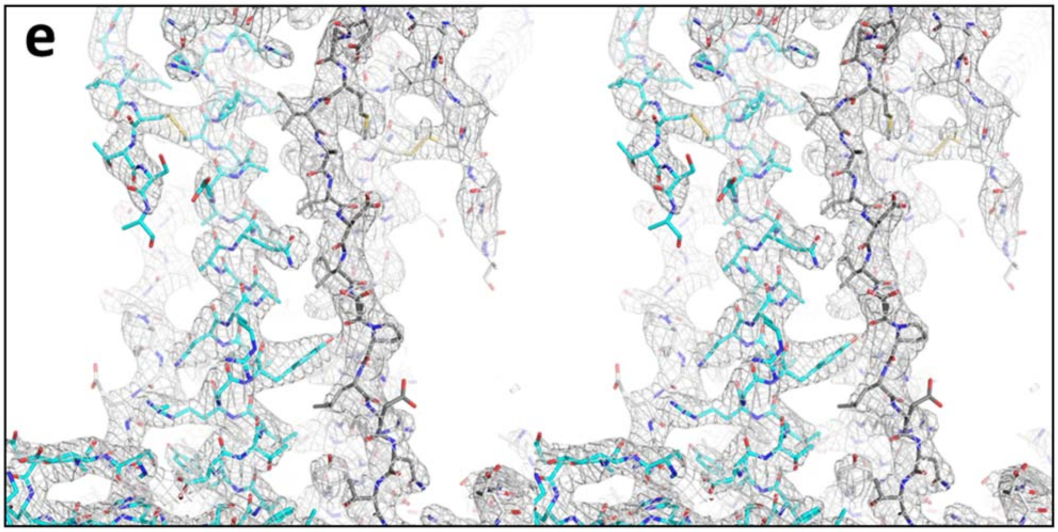

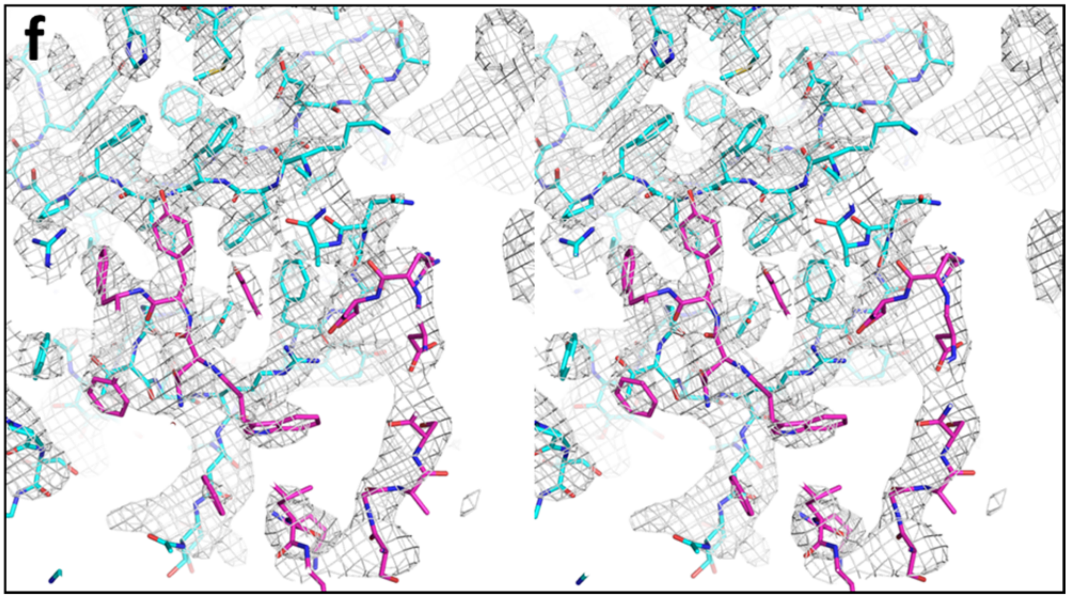

Supplementary Figure 2. Electron density maps for the ΔNgB-glyco/1G2 structure. 2|Fo|-|Fc| electron density map contoured to 2σ along: **a)** domain III helix αC; **b)** Asn208 and its linked N-acetyleglucosamine; **c)** the three-fold axis with a top view around Leu484; **d)** |Fo|-|Fc| "kicked" omit map for gB residues Phe297-Phe300 and 1G2 heavy chain residues Asn102-Phe104 contoured to 3σ; **e)** and **f)** Stereo images of 2|Fo|-|Fc| electron density maps contoured to 2σ. Electron density maps are shown as a mesh around gB residues in stick representation. All images were created in Pymol.

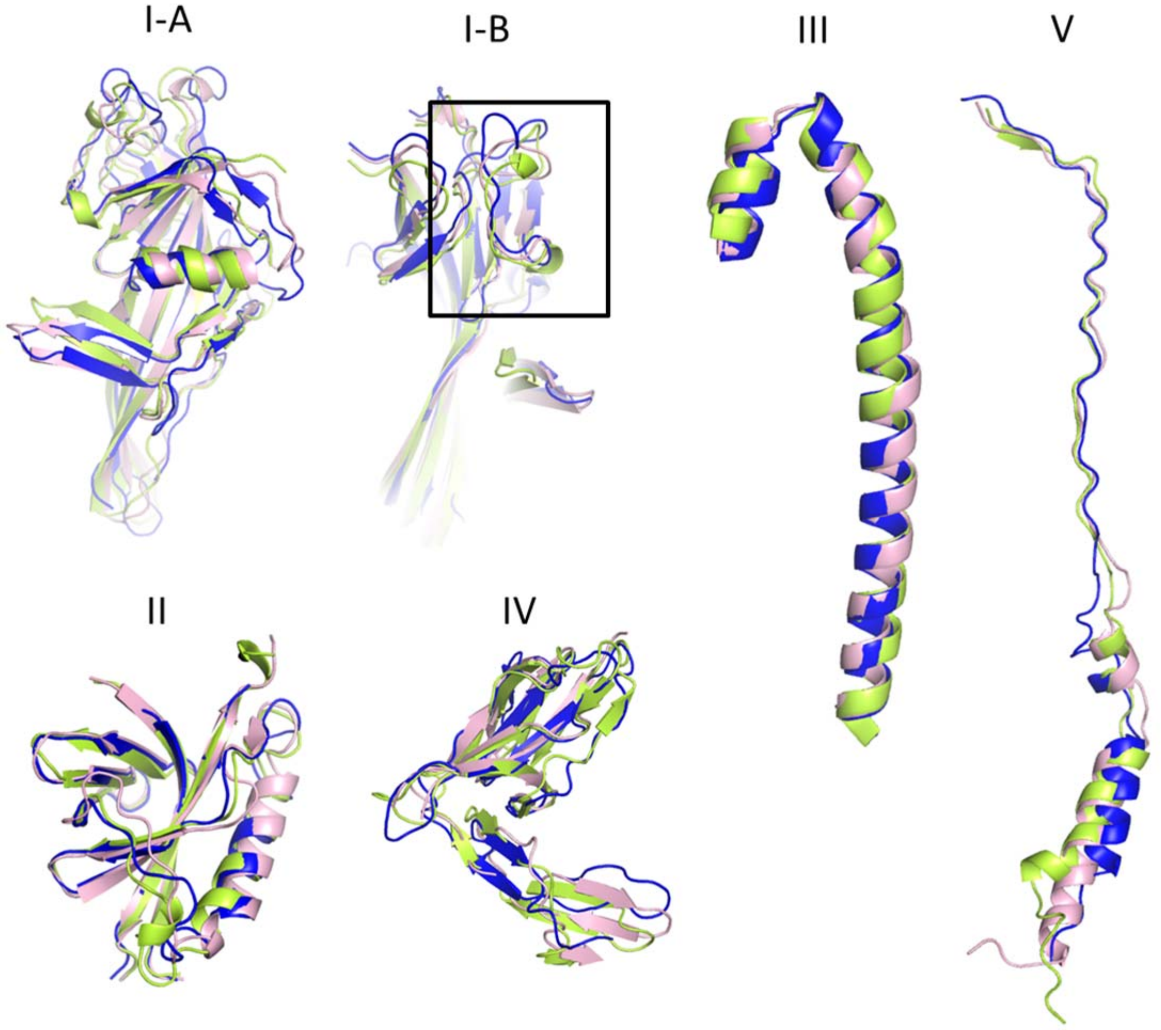

Supplementary Figure 3. Comparison of individual domains of HCMV gB with HSV and EBV gB. Individual domains of HCMV gB (I-V) were superimposed on the corresponding domains in HSV (green) or EBV gB (pink) using LSQKAB in CCP4. The view in I-B is rotated 60 degrees away from the reader from the view in I-A, with the 1G2 epitope boxed in black.

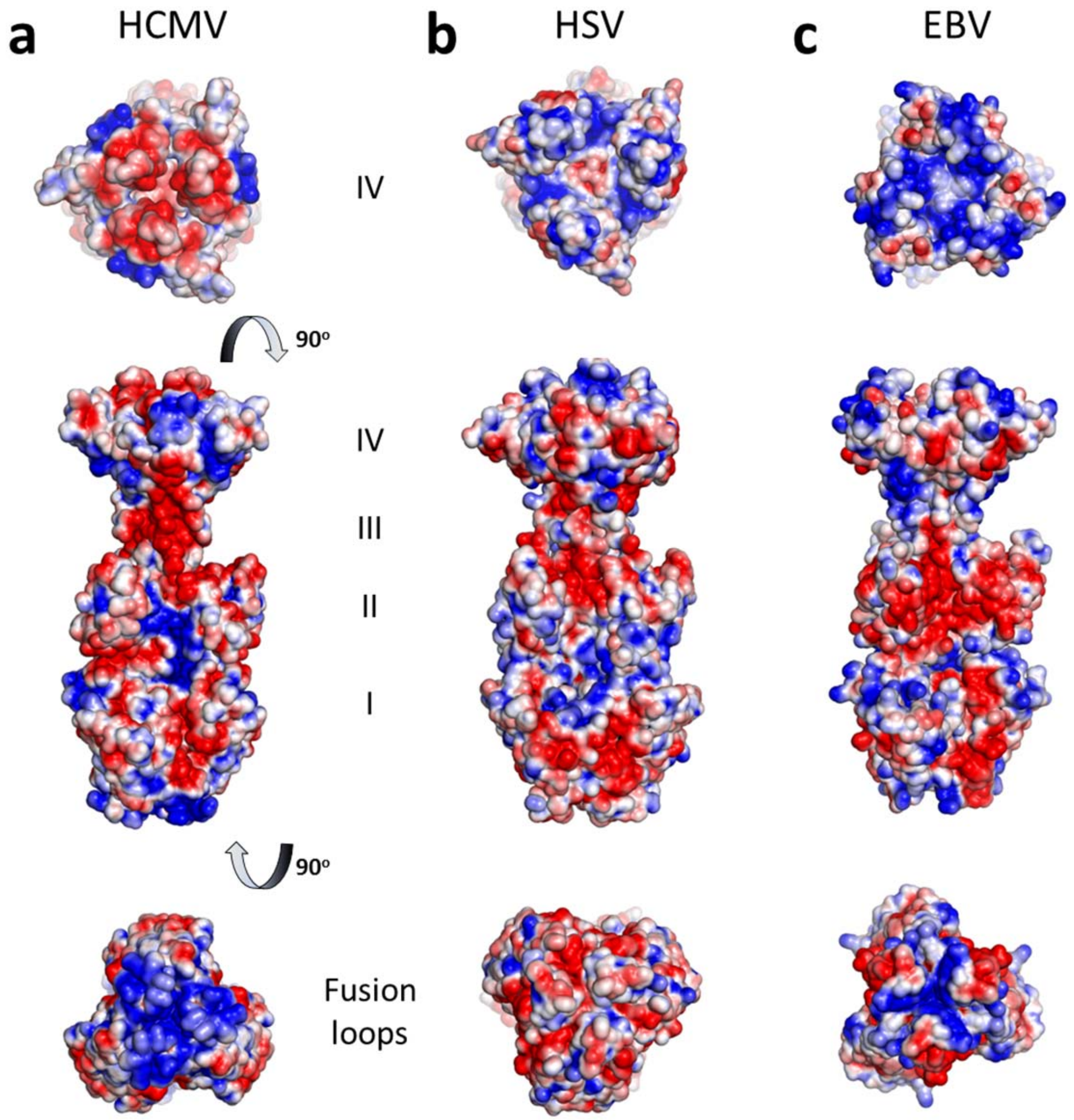

Supplementary Figure 4. Comparison of surface charge distribution of herpesvirus gB. Surface charge distribution of HCMV (a), HSV (b) and EBV (c) gB generated using PyMol, contoured to -2 to +2 *k*T/e. Blue represents positive charge and red represents negative. The top and bottom panels are views generated by rotating the view in the middle 90° towards and away from the reader, respectively.

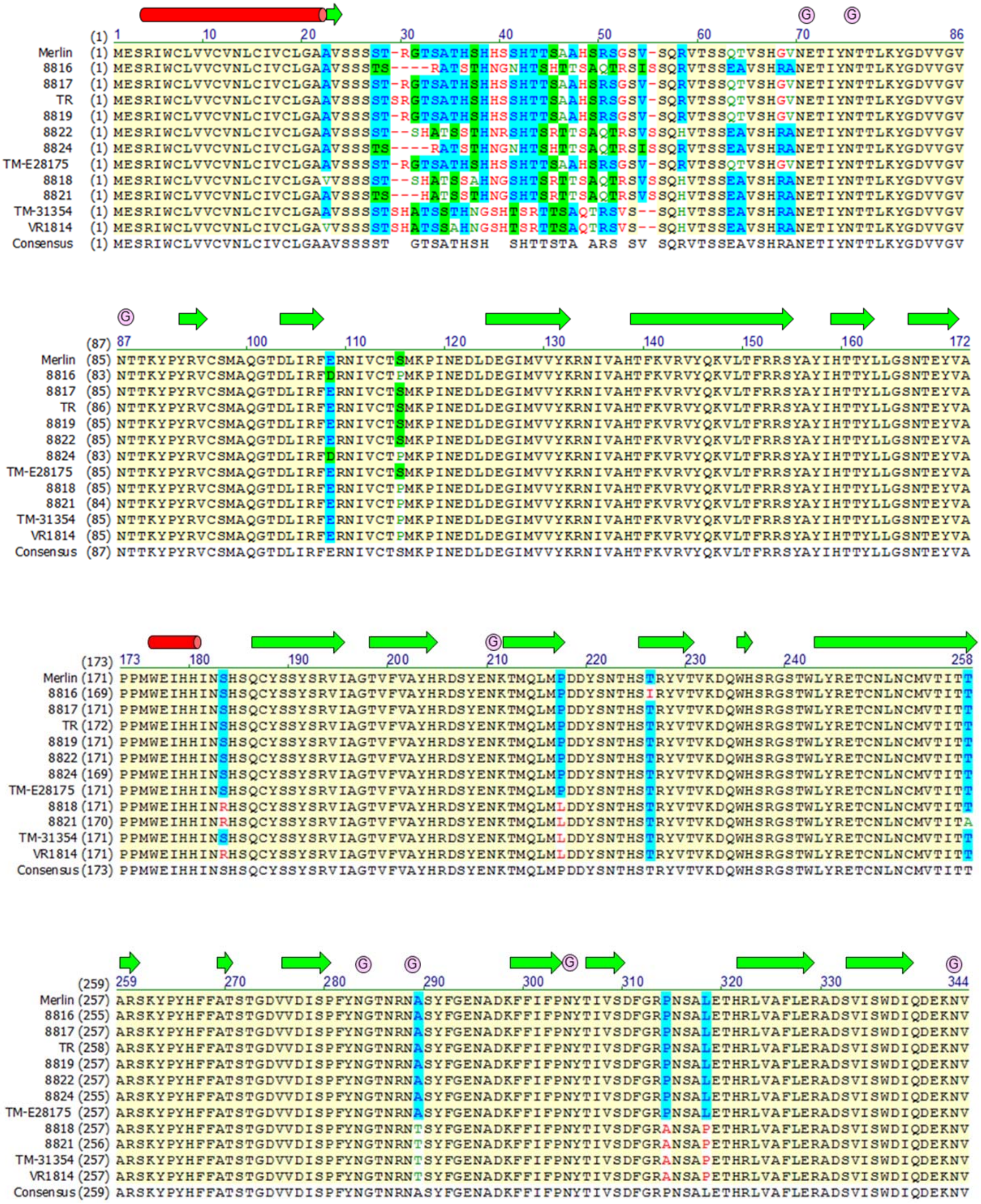
(1) 1 10 20 30 40 50 60 70 86
Merlin (1) MESRIWCLVVCVNLCIVCLGAAVSSSST-RGTSATHSHHSSHTTSAAHSRSGSV-SQRVTSSQTVSHGVNETIYNTTLKYGDVVGV
8816 (1) MESRIWCLVVCVNLCIVCLGAAVSSSTS----RATSTHNGNHTSHTTSAQTRSISSQRVTSSEAVSHRANETIYNTTLKYGDVVGV
8817 (1) MESRIWCLVVCVNLCIVCLGAAVSSSST-RGTSATHSHHSSHTTSAAHSRSGSV-SQRVTSSQTVSHGVNETIYNTTLKYGDVVGV
TR (1) MESRIWCLVVCVNLCIVCLGAAVSSSSTSRGTSATHSHHSSHTTSAAHSRSGSV-SQRVTSSQTVSHGVNETIYNTTLKYGDVVGV
8819 (1) MESRIWCLVVCVNLCIVCLGAAVSSSST-RGTSATHSHHSSHTTSAAHSRSGSV-SQRVTSSQTVSHGVNETIYNTTLKYGDVVGV
8822 (1) MESRIWCLVVCVNLCIVCLGAAVSSSST--SHATSSTHNRSHTSRTTSAQTRSVSSQHVTSSEAVSHRANETIYNTTLKYGDVVGV
8824 (1) MESRIWCLVVCVNLCIVCLGAAVSSSTS----RATSTHNGNHTSHTTSAQTRSISSQRVTSSEAVSHRANETIYNTTLKYGDVVGV
TM-E28175 (1) MESRIWCLVVCVNLCIVCLGAAVSSSST-RGTSATHSHHSSHTTSAAHSRSGSV-SQRVTSSQTVSHGVNETIYNTTLKYGDVVGV
8818 (1) MESRIWCLVVCVNLCIVCLGAVVSSSST--SHATSSAHNGSHTSRTTSAQTRSVSSQHVTSSEAVSHRANETIYNTTLKYGDVVGV
8821 (1) MESRIWCLVVCVNLCIVCLGAAVSSSTS---HATSSTHNGSHTSRTTSAQTRSVSSQHVTSSEAVSHRANETIYNTTLKYGDVVGV
TM-31354 (1) MESRIWCLVVCVNLCIVCLGAAVSSSSTSHATSSTHNGSHTSRTTSAQTRSVS--SQHVTSSEAVSHRANETIYNTTLKYGDVVGV
VR1814 (1) MESRIWCLVVCVNLCIVCLGAVVSSSSTSHATSSAHNGSHTSRTTSAQTRSVS--SQHVTSSEAVSHRANETIYNTTLKYGDVVGV
Consensus (1) MESRIWCLVVCVNLCIVCLGAAVSSSST GTSATHSH SHTTSTA ARS SV SQRVTSSEAVSHRANETIYNTTLKYGDVVGV
(87) 87 100 110 120 130 140 150 160 172
Merlin (85) NTTKYPYRVCSMAQGTDLIRFERNIVCTSMKPINEDLDEGIMVVYKRNIVAHTFKVRVYQKVLTFRRSYAYIHTTYLLGSNTEYVA
8816 (83) NTTKYPYRVCSMAQGTDLIRFDRNIVCTPMKPINEDLDEGIMVVYKRNIVAHTFKVRVYQKVLTFRRSYAYIHTTYLLGSNTEYVA
8817 (85) NTTKYPYRVCSMAQGTDLIRFERNIVCTSMKPINEDLDEGIMVVYKRNIVAHTFKVRVYQKVLTFRRSYAYIHTTYLLGSNTEYVA
TR (86) NTTKYPYRVCSMAQGTDLIRFERNIVCTSMKPINEDLDEGIMVVYKRNIVAHTFKVRVYQKVLTFRRSYAYIHTTYLLGSNTEYVA
8819 (85) NTTKYPYRVCSMAQGTDLIRFERNIVCTSMKPINEDLDEGIMVVYKRNIVAHTFKVRVYQKVLTFRRSYAYIHTTYLLGSNTEYVA
8822 (85) NTTKYPYRVCSMAQGTDLIRFERNIVCTSMKPINEDLDEGIMVVYKRNIVAHTFKVRVYQKVLTFRRSYAYIHTTYLLGSNTEYVA
8824 (83) NTTKYPYRVCSMAQGTDLIRFDRNIVCTPMKPINEDLDEGIMVVYKRNIVAHTFKVRVYQKVLTFRRSYAYIHTTYLLGSNTEYVA
TM-E28175 (85) NTTKYPYRVCSMAQGTDLIRFERNIVCTSMKPINEDLDEGIMVVYKRNIVAHTFKVRVYQKVLTFRRSYAYIHTTYLLGSNTEYVA
8818 (85) NTTKYPYRVCSMAQGTDLIRFERNIVCTPMKPINEDLDEGIMVVYKRNIVAHTFKVRVYQKVLTFRRSYAYIHTTYLLGSNTEYVA
8821 (84) NTTKYPYRVCSMAQGTDLIRFERNIVCTPMKPINEDLDEGIMVVYKRNIVAHTFKVRVYQKVLTFRRSYAYIHTTYLLGSNTEYVA
TM-31354 (85) NTTKYPYRVCSMAQGTDLIRFERNIVCTPMKPINEDLDEGIMVVYKRNIVAHTFKVRVYQKVLTFRRSYAYIHTTYLLGSNTEYVA
VR1814 (85) NTTKYPYRVCSMAQGTDLIRFERNIVCTPMKPINEDLDEGIMVVYKRNIVAHTFKVRVYQKVLTFRRSYAYIHTTYLLGSNTEYVA
Consensus (87) NTTKYPYRVCSMAQGTDLIRFERNIVCTSMKPINEDLDEGIMVVYKRNIVAHTFKVRVYQKVLTFRRSYAYIHTTYLLGSNTEYVA
(173) 173 180 190 200 210 220 230 240 258
Merlin (171) PPMWEIHHINSHSQCYSSYSRVIAGTVFVAYHRDSYENKTMQLMPDDYSNTHSTRYVTVKDQWHSRGSTWLYRETCNLNCMVTITT
8816 (169) PPMWEIHHINSHSQCYSSYSRVIAGTVFVAYHRDSYENKTMQLMPDDYSNTHSIRYVTVKDQWHSRGSTWLYRETCNLNCMVTITT
8817 (171) PPMWEIHHINSHSQCYSSYSRVIAGTVFVAYHRDSYENKTMQLMPDDYSNTHSTRYVTVKDQWHSRGSTWLYRETCNLNCMVTITT
TR (172) PPMWEIHHINSHSQCYSSYSRVIAGTVFVAYHRDSYENKTMQLMPDDYSNTHSTRYVTVKDQWHSRGSTWLYRETCNLNCMVTITT
8819 (171) PPMWEIHHINSHSQCYSSYSRVIAGTVFVAYHRDSYENKTMQLMPDDYSNTHSTRYVTVKDQWHSRGSTWLYRETCNLNCMVTITT
8822 (171) PPMWEIHHINSHSQCYSSYSRVIAGTVFVAYHRDSYENKTMQLMPDDYSNTHSTRYVTVKDQWHSRGSTWLYRETCNLNCMVTITT
8824 (169) PPMWEIHHINSHSQCYSSYSRVIAGTVFVAYHRDSYENKTMQLMPDDYSNTHSTRYVTVKDQWHSRGSTWLYRETCNLNCMVTITT
TM-E28175 (171) PPMWEIHHINSHSQCYSSYSRVIAGTVFVAYHRDSYENKTMQLMPDDYSNTHSTRYVTVKDQWHSRGSTWLYRETCNLNCMVTITT
8818 (171) PPMWEIHHINRHSQCYSSYSRVIAGTVFVAYHRDSYENKTMQLMLDDYSNTHSTRYVTVKDQWHSRGSTWLYRETCNLNCMVTITT
8821 (170) PPMWEIHHINRHSQCYSSYSRVIAGTVFVAYHRDSYENKTMQLMLDDYSNTHSTRYVTVKDQWHSRGSTWLYRETCNLNCMVTITA
TM-31354 (171) PPMWEIHHINSHSQCYSSYSRVIAGTVFVAYHRDSYENKTMQLMLDDYSNTHSTRYVTVKDQWHSRGSTWLYRETCNLNCMVTITT
VR1814 (171) PPMWEIHHINRHSQCYSSYSRVIAGTVFVAYHRDSYENKTMQLMLDDYSNTHSTRYVTVKDQWHSRGSTWLYRETCNLNCMVTITT
Consensus (173) PPMWEIHHINSHSQCYSSYSRVIAGTVFVAYHRDSYENKTMQLMPDDYSNTHSTRYVTVKDQWHSRGSTWLYRETCNLNCMVTITT
(259) 259 270 280 290 300 310 320 330 344
Merlin (257) ARSKYPYHFFATSTGDVVDISPFYNGTNRNASYFGENADKFFIFPNYTIVSDFGRPNSALETHRLVAFLERADSVISWDIQDEKNV
8816 (255) ARSKYPYHFFATSTGDVVDISPFYNGTNRNASYFGENADKFFIFPNYTIVSDFGRPNSALETHRLVAFLERADSVISWDIQDEKNV
8817 (257) ARSKYPYHFFATSTGDVVDISPFYNGTNRNASYFGENADKFFIFPNYTIVSDFGRPNSALETHRLVAFLERADSVISWDIQDEKNV
TR (258) ARSKYPYHFFATSTGDVVDISPFYNGTNRNASYFGENADKFFIFPNYTIVSDFGRPNSALETHRLVAFLERADSVISWDIQDEKNV
8819 (257) ARSKYPYHFFATSTGDVVDISPFYNGTNRNASYFGENADKFFIFPNYTIVSDFGRPNSALETHRLVAFLERADSVISWDIQDEKNV
8822 (257) ARSKYPYHFFATSTGDVVDISPFYNGTNRNASYFGENADKFFIFPNYTIVSDFGRPNSALETHRLVAFLERADSVISWDIQDEKNV
8824 (255) ARSKYPYHFFATSTGDVVDISPFYNGTNRNASYFGENADKFFIFPNYTIVSDFGRPNSALETHRLVAFLERADSVISWDIQDEKNV
TM-E28175 (257) ARSKYPYHFFATSTGDVVDISPFYNGTNRNASYFGENADKFFIFPNYTIVSDFGRPNSALETHRLVAFLERADSVISWDIQDEKNV
8818 (257) ARSKYPYHFFATSTGDVVDISPFYNGTNRNTSYFGENADKFFIFPNYTIVSDFGRANSAPETHRLVAFLERADSVISWDIQDEKNV
8821 (256) ARSKYPYHFFATSTGDVVDISPFYNGTNRNTSYFGENADKFFIFPNYTIVSDFGRANSAPETHRLVAFLERADSVISWDIQDEKNV
TM-31354 (257) ARSKYPYHFFATSTGDVVDISPFYNGTNRNTSYFGENADKFFIFPNYTIVSDFGRANSAPETHRLVAFLERADSVISWDIQDEKNV
VR1814 (257) ARSKYPYHFFATSTGDVVDISPFYNGTNRNTSYFGENADKFFIFPNYTIVSDFGRANSAPETHRLVAFLERADSVISWDIQDEKNV
Consensus (259) ARSKYPYHFFATSTGDVVDISPFYNGTNRNASYFGENADKFFIFPNYTIVSDFGRPNSALETHRLVAFLERADSVISWDIQDEKNV

Contd.

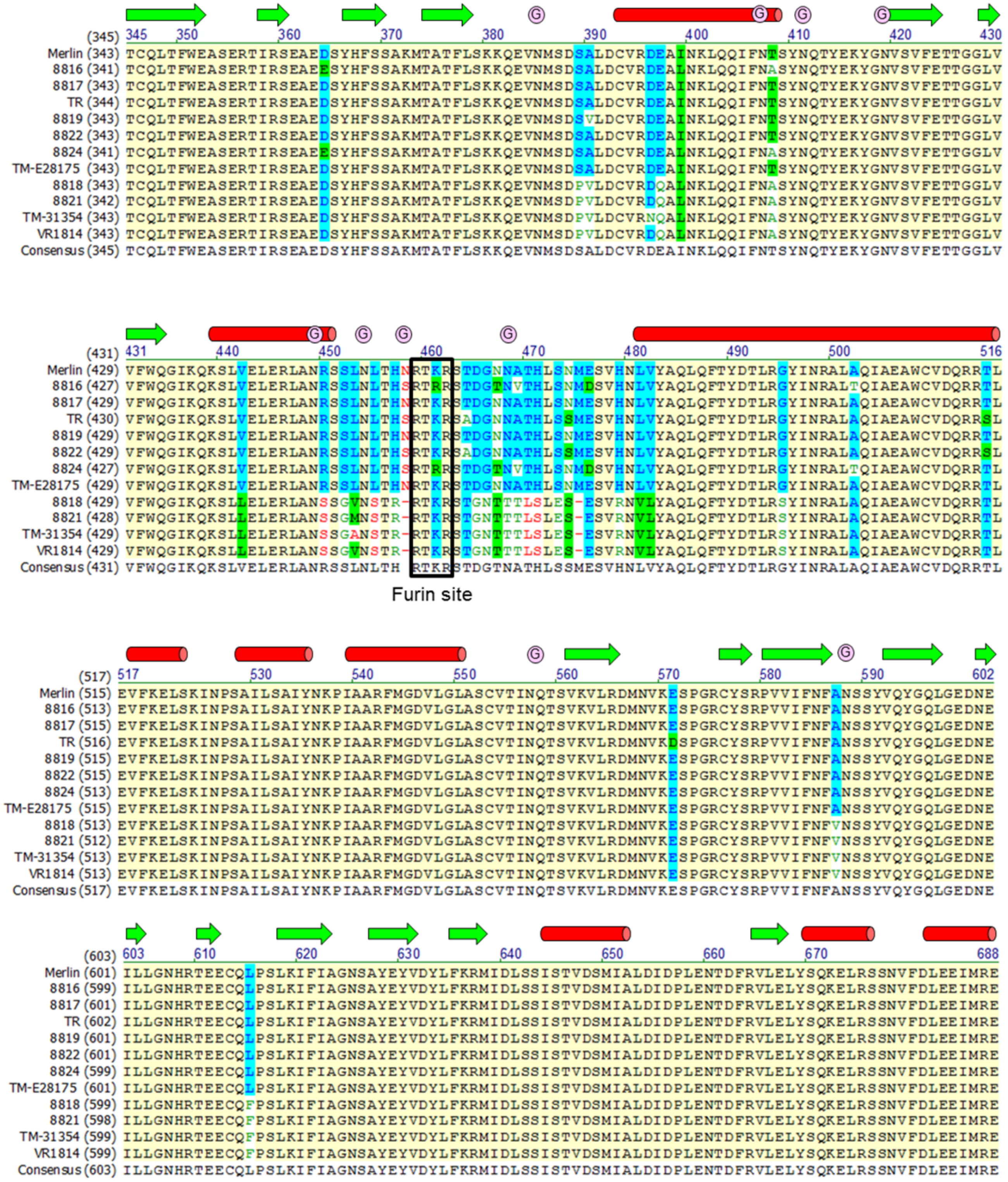
Merlin
8816
8817
TR
8819
8822
8824
TM-E28175
8818
8821
TM-31354
VR1814
Consensus (345) TCQLTFWEASERTIRSEAEDSYHFSSAKMTATFLSKKQEVNMSDSALDCVRDEAINKLQQIFNTSYNQTYEKYGNVSVFETTGGLV
Consensus (431) VFWQGIKQKSLVELERLANRSSLNLTH RTKRSTDGTNATHLSSMESVHNLVYAQLQFTYDTLRGYINRALAQIAEAWCVDQRRTL
Furin site
Consensus (517) EVFKELSKINPSAILSAIYNKPIAARFMGDVLGLASCVTINQTSVKVLRDMNVKESPGRCYSRPVVIFNFANSSYVQYGQLGEDNE
Consensus (603) ILLGNHRTEECQLPSLKIFIAGNSAYEYVDYLFKRMIDLSSISTVDSMIALDIDPLENTDFRVLELYSQKELRSSNVFDLEEIMRE

Contd.

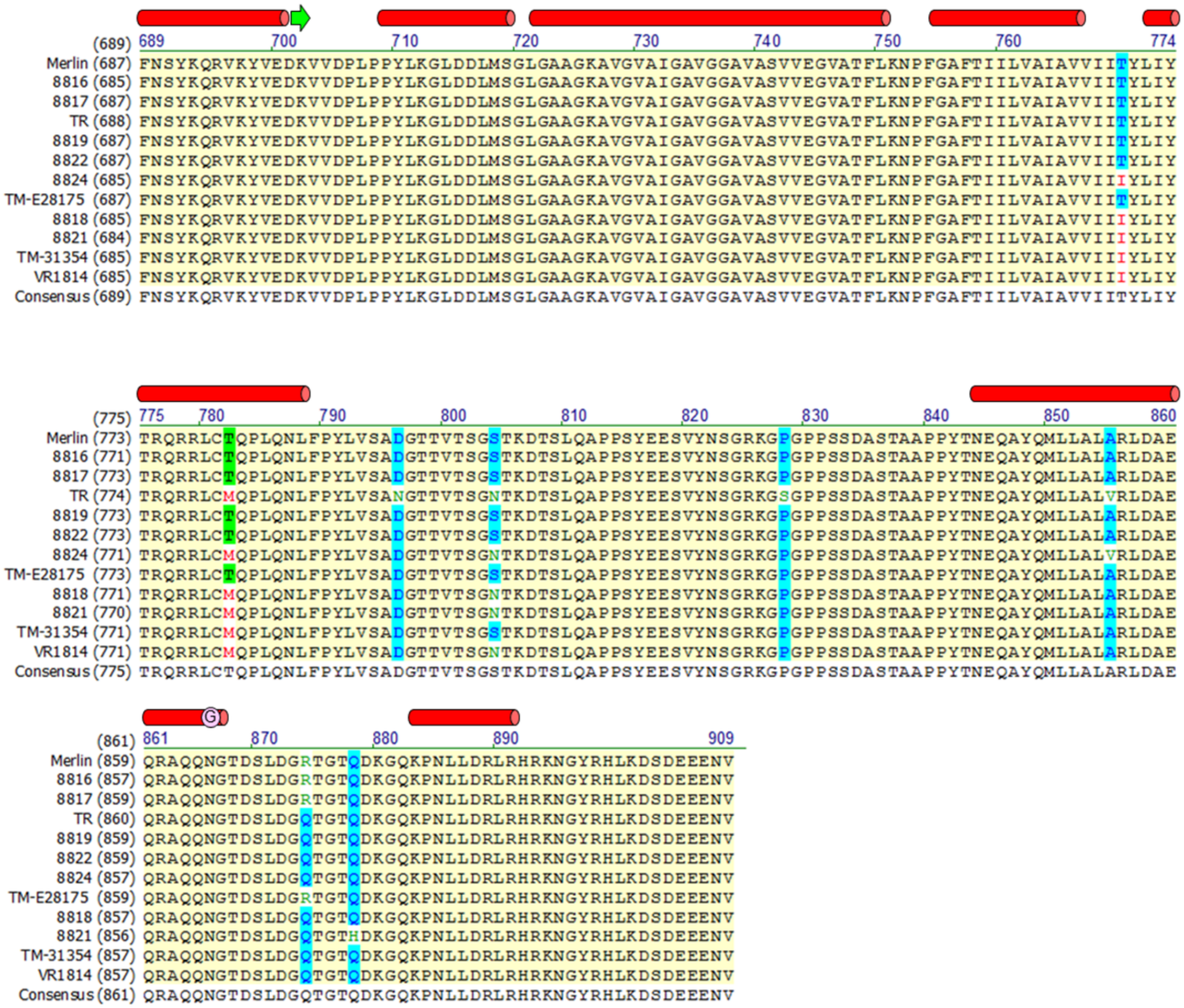

Supplementary Figure 5. Sequence alignment of gB from various HCMV strains. Full length gB sequences from 12 strains of HCMV were aligned using AlignX (Vector NTI, Invitrogen Inc.). Secondary structure elements are represented as red cylinders for helices and green arrows for beta sheets, as predicted for the Merlin strain sequence using the JPRED server (University of Dundee).

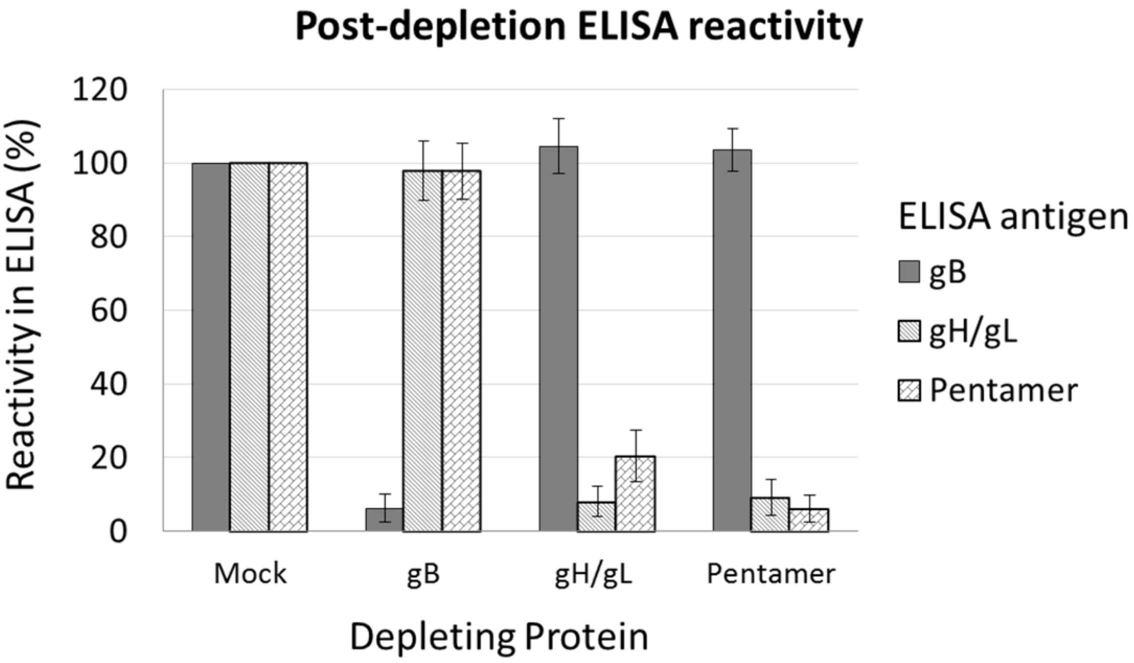

Supplementary Figure 6. Depletion efficiency and specificity of soluble gB, gH/gL and pentamer on human sera. Sera incubated with gB-698glyc, gH/gL or pentamer as described in Table 4 were tested for remaining reactivity to the respective proteins in an ELISA assay. Titers are expressed as a percentage of titers from mock-depleted sera. The x-axis represents the depleting protein. Error bars represent standard deviation across five individual serum samples.

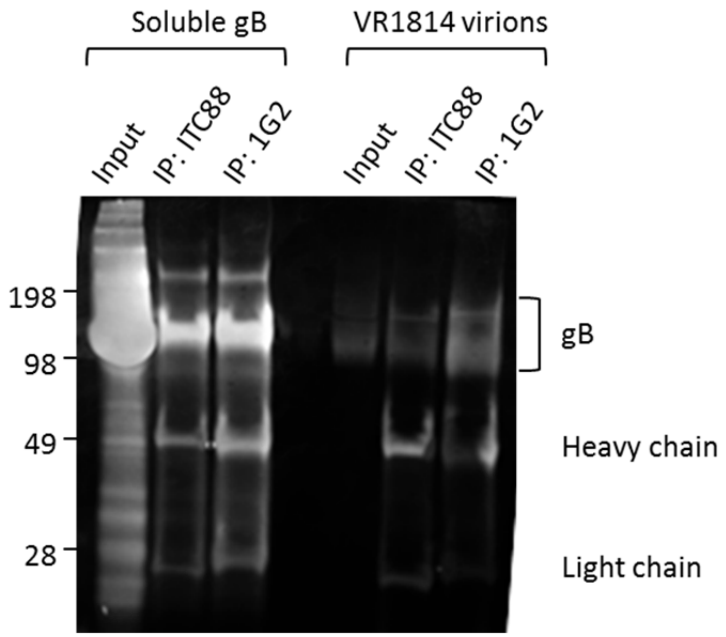

Supplementary Figure 7. Western blot for gB immunoprecipitated from purified VR1814 virions. Uncropped gel image for western blot shown in Figure 5B.

Supplementary Table 1 RMSD among herpesviruses postfusion gBs

gB Domain (residues in CMV gB)	2GUM (HSV gB)	3FVC (EBV gB)	4OSN (CMV gB AD-4 alone)	4OT1 (CMV gB AD-4 – SM5 complex)
I (134-343)*	2.96	2.679		
II (344-436)	2.7	2.3	0.983	0.754
III (479-533)	0.951	0.851		
IV (88-103, 538-638)	2.12	1.533		
V (642-695)	2.744	2.487		

* Domain I was aligned defining residue ranges 136-274, 297-303 and 321-335.

HCMV gB domains were individually superimposed onto corresponding domains from HSV or EBV gBs or to the isolated AD-4 domain structures using LSQKAB in the CCP4 suite. The boundaries of the domains are indicated in parentheses. Domain I was defined as residue ranges 136-274, 297-303 and 321-335.

Supplementary Table 2 Contact residues at the gB-1G2 interface

Residue in gB		Atom	Residue in 1G2		Atom	Distance (Å)
Tyr	280	OH	Tyr	103H	N	3.28
Gly	282	O	Asn	102H	OD1	2.64
			Tyr	103H	O	2.59
Gly	282	C	Tyr	103H	O	3.39
Thr	283	O	Asn	102H	CG	3.1
			Asn	102H	OD1	2.61
			Asn	102H	ND2	3.06
		CA	Asn	102H	OD1	3.28
		C	Asn	102H	OD1	2.9
		CG2	Phe	105H	CZ	3.47
		OG1	Tyr	50L	OH	3.5
Arg	285	CB	Trp	101H	O	3.38
		CG	Trp	101H	NE1	3.22
		CD	Trp	101H	O	3
		CZ	Ser	32H	OG	3.5
		NH1	Ser	32H	O	3.38
		NH2	Ser	32H	O	3.46
			Ser	32H	OG	2.66
Asn	286	CB	Tyr	109H	OH	3.05
		CG	Tyr	109H	OH	3.31
Phe	290	CD2	Ser	32H	OG	3.27
Gly	291	O	Arg	31H	NH1	2.95
Glu	292	O	Ser	32H	CB	3.24
Ala	294	N	Arg	31H	O	2.61
		CA	Arg	31H	O	3.39
		CB	Arg	31H	O	3.1
Ile	299	O	Tyr	103H	CE2	3.39
			Phe	104H	CE1	3.44
		N	Tyr	103H	CE2	3.12
			Tyr	103H	CZ	3.4
			Tyr	103H	OH	3.49
		CG1	Tyr	103H	CD2	3.45

Table of contact residues within a 3.5 Å cutoff was generated using the CONTACT program within the CCP4 suite.

Supplementary Table 3. Details of SPR analysis of gB-1G2 interaction

Sample		Rmax (RU)	Chi² (RU²)	Ligand Level (RU)	Max binding (RU)	Capture-adjusted max binding
gB mutants	ΔNgB-glyco	60.5	0.0582	106.2	36.4	0.34
	Y280A	260.2	0.0535	92	5.1	0.06
	N281A	41.2	0.0565	95.7	17.7	0.18
	T283A	38.4	0.0709	95.6	20.9	0.22
	N284A	75.4	0.284	220.9	50.4	0.23
	R285A	36.8	0.0905	91.8	2	0.02
	N286A	122.2	1.76	222.9	111.6	0.5
	NRN-AAA*	0.6	0.574	219.9	4.6	0.02
	F290A	34.5	0.0512	97.6	6	0.06
	E292A	39.8	0.0492	97.7	12	0.12
	N293A/D295A	81.6	0.152	224.1	45.2	0.2
	YND-AAA**	51.1	0.0498	93.8	3.5	0.04
	F297A	176.6	0.0491	97.6	9.1	0.09
	F298A	42.2	0.0512	102.1	18	0.18
	I299A	38.8	0.0614	101.6	13.3	0.13
	F298A/I299A	72	0.102	94.6	2.9	0.03
1G2	WT	25.6	0.0735	44.3	14.2	0.32
1G2 heavy chain	R31S	24.1	0.0545	34.4	12.7	0.37
	S32A	30.5	0.0922	56.3	20.5	0.36
	N102A	17.5	0.0802	55.8	9.9	0.18
	Y103A	14.9	0.0721	44.3	8.5	0.19
	F104A	20.9	0.0753	49.3	11.5	0.23
	Y103A/F104A	7.1	0.083	44.4	4.9	0.11
	Y109A	22.5	0.0776	43.6	13.1	0.3
1G2 light chain	Y50A	25.2	0.0806	54.4	15.7	0.29
	R51A	26.4	0.0784	60.2	15.8	0.26

*NRN-AAA = N284A/R285A/N286A
**YND-AAA = Y280A/N293A/D295A

Rmax, Chi^2, ligand level, max binding level and capture-adjusted max binding level for the single cycle kinetic runs reported in Table 3. While these values are reported for the 1G2 double mutant Tyr103Ala/Phe104Ala, we could not get a reliable fit for the curve for measuring the kinetic constants reported in Table 2.
